# Therapeutic targets for diabetic nephropathy identified by druggable genome mendelian randomization: the role of the gut microbiota-metabolite axis

**DOI:** 10.3389/fendo.2026.1817400

**Published:** 2026-05-08

**Authors:** Ling Niu, Rong Ma, Cuijuan Miao, Fang Liu, Boyi Li

**Affiliations:** Department of Endocrinology, The Affiliated Calmette Hospital of Kunming Medical University, The First People’s Hospital of Kunming, Kunming, Yunnan, China

**Keywords:** diabetic nephropathy, druggable genes, gut microbiota, Mendelian randomization, single-cell analysis

## Abstract

**Background:**

Diabetic nephropathy (DN), a severe complication of diabetes, is influenced by genetic, immune, and gut microbial factors but lacks targeted therapeutic strategies. Druggable genes (DGGs) present a promising avenue, yet their causal prioritization in DN and their connections to gut microbiota-metabolite mechanisms remain underexplored.

**Methods:**

This study utilized differential expression analysis to identify differentially expressed genes (DEGs), Mendelian randomization (MR) to establish causal associations with DN, and machine learning algorithms to pinpoint key genes. Blood samples were subjected to reverse transcription-quantitative polymerase chain reaction (RT-qPCR) for preliminary validation. Further validation incorporated nomogram modeling, Gene Set Enrichment Analysis (GSEA), immune infiltration analysis, molecular docking, and single-cell RNA sequencing (scRNA-seq), which included pseudotime trajectory and cell communication analysis.

**Results:**

A total of 8,913 DEGs were identified, with MR analysis revealing 1,263 genes potentially involved in DN pathogenesis and as drug targets. *FOS* and *IL12A* were selected as potential key genes, both of which were significantly downregulated in DN and validated via RT-qPCR (*p* < 0.05). The predictive nomogram model achieved an area under the curve (AUC) greater than 0.7. Functional enrichment analysis highlighted the mTOR complex 1 (mTORC1) signaling pathway. *FOS* expression correlated positively with neutrophil infiltration (r = 0.856, *p* < 0.05), while *IL12A* inversely correlated with M2 macrophage infiltration (r = –0.377, *p* < 0.05). Molecular docking revealed that *FOS* and *IL12A* may stably bind to butyrate (binding energies: –7.3 and –7.0 kcal/mol, respectively), and *IL12A* also binds to trimethylamine (binding energy: –6.4 kcal/mol). These findings were corroborated by preliminary associations with gut microbes such as Faecalibacterium prausnitzii and Lactobacillus acidophilus. scRNA-seq preliminary analysis identified proximal convoluted tubule cell 1 (PCT1) as a central cell type, exhibiting altered cell communication and differentiation trajectories, where *FOS* expression showed dynamic changes and *IL12A* remained persistently downregulated.

**Conclusion:**

This druggable gene-oriented MR strategy preliminary nominates *FOS* and *IL12A* as potential therapeutic targets for DN. Their involvement in DN pathogenesis is mediated via mTORC1 signaling, PCT1 cellular reprogramming, immune microenvironment remodeling, and interactions with a gut microbiota-metabolite axis, laying the groundwork for targeted therapies and microecological interventions in DN.

## 1 Introduction

Diabetic nephropathy (DN) is one of the most severe microvascular complications of type 2 diabetes mellitus (T2DM) (1, 2). Approximately 40% of patients with T2DM develop DN, which has become the leading cause of end-stage renal disease (ESRD) (3, 4). The global prevalence of diabetes-related ESRD increased from 19.0% in 2000 to 29.7% in 2015, with the proportion of ESRD cases attributed to diabetes rising from 22.1% to 31.3% (5). With the ongoing rise in obesity and T2DM prevalence, the burden of chronic kidney disease due to DN is growing rapidly, significantly impacting patients’ quality of life and placing considerable strain on healthcare systems (6). Current first-line pharmacological treatments for DN, such as angiotensin-converting enzyme inhibitors and sodium-glucose cotransporter-2 inhibitors, can slow disease progression but fail to completely prevent renal injury. Additionally, their effectiveness is limited in some patients due to suboptimal responses or resistance, highlighting the urgent need for novel therapeutic targets with definitive causal evidence (7).

Druggable genes, which encode proteins with domains capable of specific drug binding, show a markedly higher success rate in drug development than non-druggable genes. Over 80% of globally approved therapeutic agents target proteins from druggable gene families (8). Focusing on druggable genes for DN target discovery could significantly accelerate development timelines and reduce failure rates. The main challenge, however, is to accurately identify those druggable genes that have a causal role in DN. Mendelian Randomization (MR), with its unique causal inference capabilities, holds promise in addressing this critical issue.

MR is a robust causal inference method that leverages genetic variants (e.g., single-nucleotide polymorphisms, SNPs) as instrumental variables (IVs) to investigate exposures. Because genetic variants are randomly inherited during meiosis and are typically unaffected by postnatal environmental factors, MR effectively mitigates confounding biases and reverse causation, common in traditional observational studies (9). Recently, MR has been widely applied to uncover the etiology of chronic diseases and validate therapeutic targets. A notable example is MR’s role in confirming the causal link between low-density lipoprotein cholesterol and coronary heart disease, providing genetic evidence to support statin therapy (10). Integrating MR with the druggable genome framework offers a promising approach for identifying genetically validated, druggable targets for DN, forming a novel theoretical foundation for its precise treatment.

Additionally, gut microbiota dysbiosis plays a pivotal role in modulating host inflammation and metabolic dysfunction (11). The gut microbiota-metabolite axis has emerged as an important factor in DN pathogenesis. Patients with DN show significant gut dysbiosis, marked by a decrease in beneficial bacteria such as Faecalibacterium and an increase in potentially harmful bacteria like Enterobacteriaceae (12, 13). Concurrent metabolite imbalances, including reduced levels of short-chain fatty acids (SCFAs) and elevated trimethylamine N-oxide (TMAO), are also linked to renal injury (14, 15). However, causal relationships between most gut microbial features and metabolites with DN remain unclear, and a comprehensive analysis of their interactions with druggable targets is lacking (16).

Building on this rationale, the present study seeks to integrate large-scale genomic, gut microbiomic, and metabolomic data. A druggable gene-based MR approach will be employed to systematically identify druggable therapeutic targets with causal links to DN, while also pinpointing key gut microbiota and metabolites that regulate DN progression. The results of this study are expected to provide new insights into the mechanisms of DN and establish a vital foundation for developing novel therapeutics and precision prevention strategies.

## 2 Data sources and methods

The overall analytical pipeline of this study is as follows: First, relevant bulk transcriptome and single-cell sequencing data were retrieved from the GEO database, followed by data preprocessing and standardization. Differentially expressed genes (DEGs) and MR-DGGs were identified through differential expression and MR analysis, respectively. Next, key candidate genes were selected using MR analysis combined with machine learning techniques, and their expression patterns were validated in an independent cohort. A nomogram model was then constructed based on these key genes, followed by Gene Set Enrichment Analysis (GSEA), immune infiltration analysis, and competitive endogenous RNA (ceRNA) network construction. Molecular docking analysis was performed to assess the interactions between gut metabolites and the key genes. Finally, single-cell RNA sequencing data were analyzed to examine gene expression across different cell types, and pseudotime, functional enrichment, and cell–cell communication analyses were conducted to explore their potential biological functions (**Supplementary Figure 1**).

### 2.1 Data sources

The training set, validation set, single-cell datasets, druggable genes, and gut microbiota targets used in this study for DN were all sourced from public databases or published literature. When selecting datasets, priority was given to excluding those unrelated to DN or with missing data. High-quality datasets with complete reference information and an impact factor greater than 6 were selected. Only datasets with standardized data and a sample size of three or more were included. Specifically, dataset GSE96804 was downloaded from the GEO database (https://www.ncbi.nlm.nih.gov/geo/) and used as the training set (17, 18). This dataset, generated by microarray sequencing (Platform GPL17586), was processed by converting probe IDs to gene symbols using the corresponding annotation file. Based on grouping information, 41 DN tissue samples and 20 healthy control tissue samples were selected. Dataset GSE104948 (19), also from GEO, served as the validation set. This microarray dataset (Platform GPL22945) was processed similarly, and 7 DN tissue samples and 3 control samples were selected for external validation of the training set results.

The single-cell dataset was constructed by integrating four relevant datasets from GEO. GSE151302 contributed 5 healthy kidney control samples (20); GSE131882 included 3 DN samples and 3 healthy controls (21); GSE131685 provided 3 DN kidney samples (22); and GSE195460 added 5 DN kidney samples and 5 healthy controls (23). After integration, the final single-cell cohort comprised 11 DN kidney samples and 13 healthy kidney control samples.

Druggable genes were sourced from the supplementary tables of a published study (24). After deduplication, 5,883 non-redundant genes were retained for subsequent screening of potential therapeutic targets. Relationships between gut microbiota targets and their corresponding metabolites were retrieved from the gutMGene database (http://bio-computing.hrbmu.edu.cn/gutmgene) (25).

### 2.2 Differential expression analysis between DN and control samples

To identify potential DEGs between DN and control samples, differential expression analysis was performed on the training set by comparing DN tissues to normal controls (DN vs. Control) using the “limma” R package. The analysis was conducted with Benjamini-Hochberg correction, and DEGs were defined based on a significance threshold of raw *p*-value < 0.05 (26). The results were visualized using the “ggplot2” package (version 3.5.2) to generate a volcano plot and the “ComplexHeatmap” package (version 2.20.0) (27) to create a heatmap.

### 2.3 Mendelian randomization analysis

Expression quantitative trait loci (eQTL) data for DEGs were used as exposures, and summary statistics for DN (trait ID: ebi-a-GCST90018832, N = 452,280) were obtained from the GWAS database. The “TwoSampleMR” R package was employed to select IVs based on the following criteria: genome-wide significance (*p* < 5 × 10^−6^), an F-statistic > 10, and a minimum of three independent (LD-clumped) SNPs. After harmonizing exposure and outcome data, MR analyses were performed using five methods, including inverse-variance weighted (IVW). The IVW method weights the effect size and standard error of each IV. For this exploratory screening of drug targets, genes with an IVW *p*-value < 0.05 were considered potential causal candidates (MR-DGGs). To reduce the risk of false positives, sensitivity analyses were conducted on MR-DGGs to assess result heterogeneity. A Q *p*-value greater than 0.05 indicated no significant heterogeneity. Horizontal pleiotropy was evaluated using Cochran’s Q test (*p* > 0.05). The robustness of the results was further validated using Leave-one-out analysis and Steiger analysis, with TRUE & *p* < 0.05 suggesting a unidirectional causal relationship.

### 2.4 Candidate gene identification and enrichment analysis

To identify candidate genes relevant to DN from the MR-DGGs, the “VennDiagram” R package (version 1.7.3) was used. The intersection between the up-regulated DEGs from the transcriptomic analysis and MR-DGGs exhibiting an odds ratio (OR) greater than 1 was considered. Similarly, the intersection of down-regulated DEGs with MR-DGGs exhibiting an OR less than 1 was analyzed. The union of these two intersecting gene sets was then designated as the final candidate gene set.

To investigate the biological functions and signaling pathways associated with these candidate genes, Gene Ontology (GO) and Kyoto Encyclopedia of Genes and Genomes (KEGG) pathway enrichment analyses were performed using the “clusterProfiler” R package (version 4.12.0) (28). A significance threshold of raw *p*-value < 0.05 was applied to identify enriched terms and pathways. The top 15 pathways, corrected by Benjamini-Hochberg, were visualized.

### 2.5 Identification of candidate gut microbiota-targeted genes

To identify genes within the candidate set that are known targets of the human gut microbiota, the “VennDiagram” R package was used to find the intersection between our candidate genes and a curated list of gut microbiota-targeted genes from existing studies. The resulting genes were defined as candidate gut microbiota-targeted genes. Additionally, MR analysis was conducted specifically on these candidate gut microbiota-targeted genes.

### 2.6 Key feature gene selection

Feature genes in the DN training set were selected through LASSO regression analysis using the “glmnet” R package (version 4.1-8) (29), based on the gene expression matrix of candidate gut microbiota targets. SVM-RFE analysis was then performed across two disease training sets using the “caret” R package (version 6.0-93) on the same expression matrix. The intersection of genes identified by both machine learning methods was used to define key signature genes.

### 2.7 Expression validation of key feature genes

To further assess and validate the expression patterns of these key genes in the disease context, their expression levels were compared between disease and normal control samples in both the training and validation sets. Boxplots generated with the “ggplot2” package visualized the results. The final key genes, characterized by statistically significant and directionally consistent differential expression, were used as the foundation for subsequent analyses.

### 2.8 Nomogram construction and evaluation

To evaluate the predictive performance of the key genes, a nomogram was developed based on their expression in the training set (GSE96804) using the “rms” R package (version 8.0-0) (https://github.com/harrelfe/rms). A total score was calculated by summing the scores assigned to each gene, with the probability of disease occurrence inferred accordingly (higher scores indicate a greater risk). During model construction, rigorous internal validation was performed: calibration curves were plotted using the Bootstrap method with 120 repetitions (Bootstrap = 120) in the “regplot” package (version 1.1) (https://CRAN.R-project.org/package=regplot), and the Hosmer-Lemeshow test was applied to evaluate model fit, with a *p*-value greater than 0.05 indicating a good fit. Additionally, the model’s reliability and potential clinical utility were assessed by plotting ROC curves and decision curves using the “pROC” package (version 1.19.0.1) (30) and the “rmda” package (version 1.6) (31).

### 2.9 Gene set enrichment analysis

To elucidate the potential biological functions and pathways of key genes in DN, Spearman correlation coefficients between each key gene and all other genes were calculated for the two training datasets using the “psych” R package (version 2.4.3) (32). Gene lists were ranked based on descending correlation coefficients. GSEA was then performed using the “clusterProfiler” R package, with the “h.all.v2023.2.Hs.symbols.gmt” gene set as the reference. Pathways significantly enriched with a raw *p*-value < 0.05 were selected, and the top 10 pathways, corrected by Benjamini-Hochberg, were visualized.

### 2.10 Immune infiltration and correlation analysis

To assess immune status differences in DN, the infiltration scores of 22 immune cell types in patients with DN and normal controls were calculated in the training set using the “IOBR” R package (version 0.99.0) (33) combined with the CIBERSORT algorithm. A heatmap was generated using the “pheatmap” package (version 1.0.12) (https://github.com/raivokolde/pheatmap). Differential immune cells were identified using the Wilcoxon rank-sum test (raw *p* < 0.05), with multiple comparisons adjusted by the Benjamini-Hochberg correction. In the DN training set, Spearman correlation analysis was performed using the “psych” package to evaluate correlations among differential immune cells, as well as between key genes and differential immune cells (*p* < 0.05 and |cor| > 0.3). Correlation heatmaps were then generated for visualization.

### 2.11 Construction of key gene regulatory network

To explore the potential regulatory relationships of key genes, a ceRNA network, specifically a lncRNA–miRNA–mRNA network, was constructed. miRNAs targeting key genes (mRNAs) were retrieved from the miRDB database, and upstream long non-coding RNAs (lncRNAs) that potentially bind these miRNAs were predicted using the starBase database. The interactions among these lncRNAs, miRNAs, and key mRNAs were integrated and visualized as a regulatory network using Cytoscape software.

### 2.12 Identification of key gut microbiota and metabolites

To further investigate potential associations between the key genes and gut microbial metabolites, relationships linking key gene targets to specific gut microbiota and metabolites were obtained from the gutMGene database. A tripartite network representing “key gene–key gut microbiota–gut metabolite” interactions was constructed and visualized using Cytoscape.

### 2.13 Molecular docking

To assess potential interactions between key gene-encoded proteins (receptors) and gut metabolites (ligands), blind molecular docking was conducted using the CB-Dock2 online platform (https://cadd.labshare.cn/cb-dock2/php/blinddock.php). Gut metabolites were imported into the PubChem database (https://pubchem.ncbi.nlm.nih.gov/, August 2025) to obtain their 3D structures. The proteins encoded by the key genes were imported into the UniProt database (https://www.uniprot.org/, August 2025) and the PDB database (https://www.rcsb.org/, August 2025) to obtain the receptor 3D structures. The receptor structure with the highest resolution was selected for molecular docking simulation. Molecular docking was then performed using the CB-Dock2 platform with default parameters (number of docking cavities = 5), and the binding energy was evaluated using AutoDock Vina’s scoring function (binding energy < –5.3 kcal/mol indicates relative stability). Additionally, molecular docking simulations were conducted using the SwissDock platform with its default parameters (sampling exhaustivity = 4), and binding energy was assessed using AutoDock Vina’s scoring function (binding energy < –5.3 kcal/mol was considered relatively stable).

### 2.14 Single-cell data analysis

To further investigate gene expression patterns and regulatory mechanisms, preliminary quality control was performed on all samples in the single-cell dataset using the PercentageFeatureSet function from the “Seurat” R package (version 5.1.0) (34). Specifically, cells with fewer than 200 or more than 5,000 detected genes were excluded, as were cells with total gene expression counts below 200 or above 20,000. Additionally, cells with a mitochondrial gene proportion exceeding 10% were removed. Highly variable genes (HVGs), reflecting biological heterogeneity across cells, were identified using the FindVariableFeatures function in Seurat to minimize noise from irrelevant genes. The top 2,000 HVGs were selected for normalization. Principal component analysis (PCA) was then conducted using JackStraw and ScoreJackStraw, followed by unsupervised clustering with FindNeighbors and FindClusters (resolution = 0.4), which classified all cells into distinct clusters. Cell type annotation was performed using the FindAllMarkers function, in combination with known marker genes, to assign clusters to specific cell types (**Table 1**).

**Table 1:** Marker genes for cell annotation.

Cell types	Genes
PCT1	“CUBN”, “LRP2”, “SLC22A6”, “SLC34A1”, ”FTCD”
DCT	“SLC12A3”, “TRPM6”, “WNK4”, “KLHL3”
TAL	“UMOD”, “SLC12A1”, “CLDN16”
CNT_PC	“AQP2”, “SCNN1A”, “HSD11B2”
ICA	“AQP6”, “ATP6V0A4”, “RHBG”, “FOXI1”
ICB	“CA8”, “SLC4A9”, “GRB10”
EC	“PECAM1”, “EGFL7”, “ENG”, “FLT1”
PEC	“CLDN1”, ”WT1”, “COL4A1”
PODO	“NPHS1”, “NPHS2”, “PODXL”
VSMC	“ACTA2”, “CALD1”, “MYLK”, “TPM1”
Mac	“CD74”, “HLA-DRB1”, “HLA-DRA”
PCT2	“MT1F”, “GSTA1”, “APOE”

To assess differences in cell abundance between DN and control samples, chi-square tests were conducted using the “reshape2” package (version 1.4.4) (35), with a significance threshold of *p* < 0.05. Cells showing significant differences in abundance between DN and control groups were defined as differential cells. Wilcoxon tests were then applied to these differential cells to further analyze the expression patterns of key genes, identifying cell types with significant gene expression differences between groups (*p* < 0.05). Results were visualized using UMAP and boxplots generated with ggplot2. Cells showing both significant abundance differences and differential expression of key genes were designated as key cells.

Finally, intercellular communication between key cells and other cell populations was investigated using the “CellChat” R package (version 1.4.0) (36). The ligand-receptor database (CellChatDB.human) was imported to construct communication matrices. Both the number and strength of interactions between key cells and other subpopulations were analyzed, revealing potential signaling pathways and providing insights into the complex biological processes and mechanisms underlying DN.

### 2.15 RT-qPCR

Blood samples were collected from 10 patients with DN and 10 healthy controls. The study received approval from the Ethics Committee of Kunming First People’s Hospital (Ethics Approval No.: 2026-012-01). Total RNA was extracted using TRIzol reagent (Life Technologies, 15596018) and reverse-transcribed with the PrimeScript RT Reagent Kit (Takara, RR037A). Quantitative reverse transcription PCR (RT-qPCR) was performed on the QuantStudio™ 5 Real-Time PCR System (Thermo Fisher) using the TB Green Premix Ex Taq II Kit (Takara, RR820A). Results were presented as linearized Ct values normalized to the GAPDH gene and the indicated reference value (2^-ΔΔCt^). Primer sequences are provided in **Table 2**. Statistical analyses were conducted using GraphPad Prism (Version 8.0), and data are presented as mean ± SD with statistical significance assessed by two-tailed Student’s t-test.

**Table 2:** Primer sequences.

Genes	Sequences
hFOS-F	GCCTCTCTTACTACCACTCACC
hFOS-R	AGATGGCAGTGACCGTGGGAAT
hIL12A-F	CTCCCAAAACCTGCTGAGGG
hIL12A-R	TCCAATGGTAAACAGGCCTCC
hGAPDH-F	TGATGACATCAAGAAGGTGGTG
hGAPDH-R	ACCCTGTTGCTGTAGCCAAAT

## 3 Results

### 3.1 DEGs and MR-based integrated screening of diabetic nephropathy candidate genes

**Figure 1:**
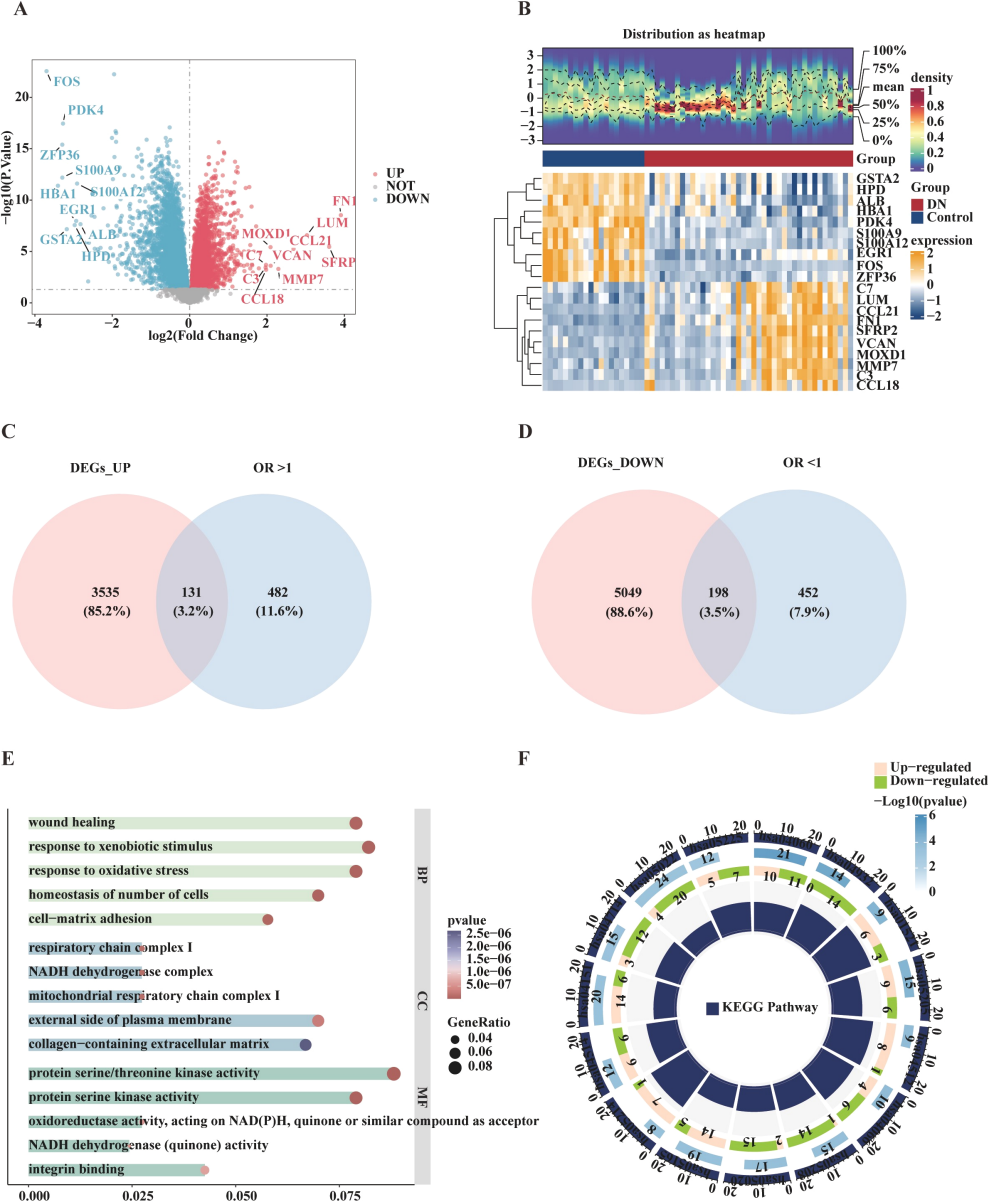
Analysis and functional enrichment of differentially expressed genes (DEGs) between the diabetic kidney disease group and the control group. **(A)** Volcano plot of DEGs associated with diabetic kidney disease. Red dots represent upregulated genes, blue dots represent downregulated genes. The top 10 most significantly up- and down-regulated genes are labeled with their gene names. **(B)** Heatmap of DEGs associated with diabetic kidney disease. The upper section displays a density heatmap of expression levels for the top 10 up- and down-regulated genes across samples, showing five quantiles and a line for the mean value. The lower section presents an expression heatmap of the top 10 up- and down-regulated genes across samples, where yellow indicates samples with high gene expression and blue indicates samples with low gene expression. **(C)** Venn diagram illustrating the overlap between upregulated DEGs and genes with an odds ratio (OR) > 1. **(D)** Venn diagram illustrating the overlap between downregulated DEGs (DEGs_DOWN) and genes with an odds ratio (OR) < 1. **(E)** Bar plot of Gene Ontology (GO) enrichment analysis. The x-axis represents enrichment significance (-log_10_(P value)), and the names of the respective GO terms are annotated. **(F)** Circular plot of KEGG pathway enrichment. The inner ring displays the association between genes and pathways (pink represents upregulated genes, green represents downregulated genes). The outer ring shows different KEGG pathways, with color gradient (from light blue to dark blue) indicating enrichment significance (-Log_10_(Pvalue)). The center of the plot includes annotations for gene ratio and other relevant information.

Differential expression analysis (DN vs. Control, *p* < 0.05) of the DN training set revealed 8,913 DEGs, comprising 3,666 up-regulated and 5,247 down-regulated genes (**Supplementary Tables 2**, **3**). Core DEGs with the most significant expression changes were visualized using a volcano plot, and the distinct expression patterns of 20 core DEGs between the two groups were further validated via heatmap analysis (**Figures 1A, B**), confirming the reliability of the DEG screening results.

Next, MR analysis was conducted using 4,064 druggable genes with available eQTL data as exposures and DN GWAS data (trait ID: ebi-a-GCST90018832) as the outcome. The IVW method was applied as the primary approach (*p* < 0.05), identifying 1,263 MR-DGGs potentially causally associated with DN (**Supplementary Table 4**). Validation through Cochran’s Q test (heterogeneity, *p* > 0.05), Steiger directionality test (*p* < 0.05), and MR-Egger intercept test (horizontal pleiotropy, *p* > 0.05) indicated no significant heterogeneity or directional pleiotropy, further supporting potential causal inference (**Supplementary Tables 5**–**7**).

To identify core candidate genes related to DN, the intersection between up-regulated DEGs and MR-DGGs with an OR > 1, as well as between down-regulated DEGs and MR-DGGs with an OR < 1, was analyzed, resulting in 329 candidate genes (**Figures 1C, D**, **Supplementary Table 8**). Functional enrichment analysis (*p* < 0.05) was then performed. GO analysis revealed 1,213 significant terms (**Figure 1E**). Biological process (BP) terms were enriched in oxidative stress response and cell population homeostasis (**Supplementary Table 9**). Cellular component (CC) terms were primarily related to mitochondrial respiratory chain complex I and collagen-containing extracellular matrix (**Supplementary Table 10**). Molecular function (MF) terms were associated with protein serine/threonine kinase activity and integrin binding (**Supplementary Table 11**). KEGG pathway analysis identified 58 enriched pathways (**Supplementary Table 12**). The top 15 core pathways included cytokine–cytokine receptor interaction, HIF-1 signaling, PI3K-Akt signaling, and others closely related to DN pathogenesis (**Figure 1F**). These results suggest that the candidate genes may contribute to DN development and progression by regulating key processes such as the inflammatory response, energy metabolism, and extracellular matrix remodeling.

### 3.2 Screening of candidate gut microbiota target genes and validation via MR analysis

**Figure 2:**
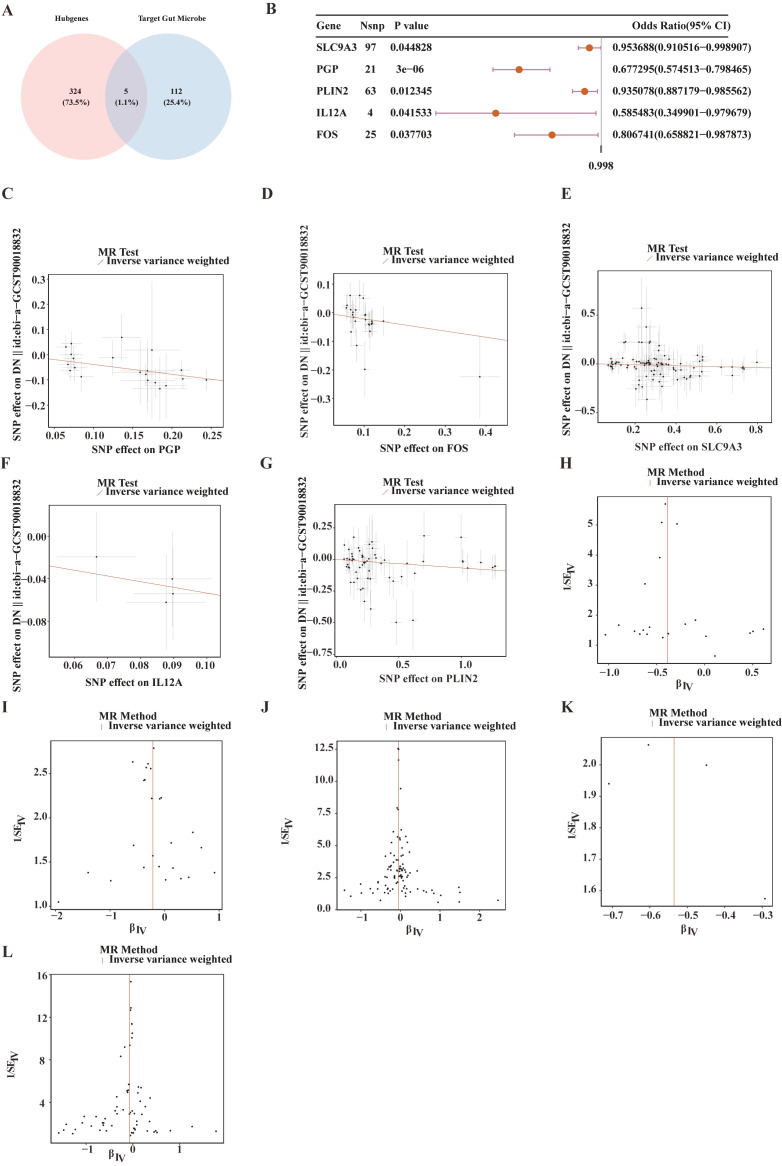
Mendelian randomization (MR) analysis of five candidate gut microbiota-targeted genes. **(A)** Venn diagram illustrating the overlap between Hub genes and target gut microbes, with numerical values indicating the gene count and proportion. **(B)** Forest plot of the Mendelian randomization analysis for the five candidate gut microbiota-targeted genes. **(C–G)** Scatter plots showing the association of the five candidate gut microbiota-targeted genes—PGP, *FOS*, SLC9A3, *IL12A*, and PLIN2—with ND, respectively. **(H–L)** Funnel plot of the association between five candidate gut microbiota-targeted genes—PGP, *FOS*, SLC9A3, *IL12A*, and PLIN2—with ND, respectively.

The intersection of the 329 candidate genes with 117 previously reported human gut microbiota target genes identified five candidate gut microbiota target genes: *PGP*, *FOS*, *SLC9A3*, *IL12A*, and *PLIN2* (**Figure 2A**). MR analysis using the IVW method indicated that all five genes were negatively associated with the risk of DN (**Figures 2B–G**). Funnel plot analysis confirmed that no single SNP significantly influenced the causal estimates, supporting the stability of the MR results (**Figures 2H–L**). Cochran’s Q test revealed no significant heterogeneity (**Supplementary Table 13**), and sensitivity analysis indicated no horizontal pleiotropy (**Supplementary Table 14**). The Steiger directionality test confirmed the direction of the causal relationship with no reverse causality detected (**Supplementary Table 15**), collectively reinforcing the robustness of the MR findings.

### 3.3 Identification of key signature genes, construction of a predictive model, and functional mechanism exploration

**Figure 3:**
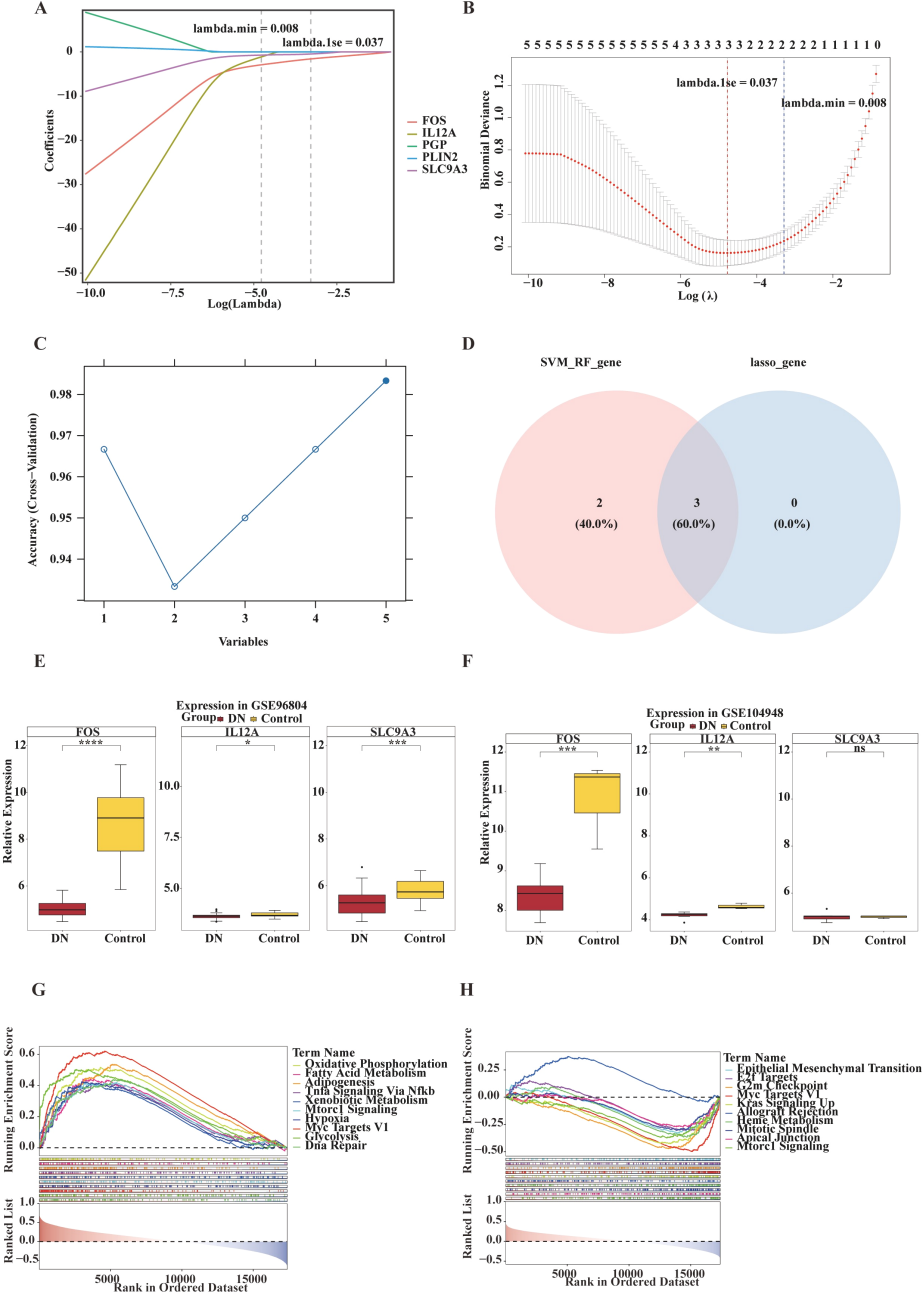
Identification of characteristic genes for diabetic nephropathy (DN) using machine learning approaches. **(A)** Profile plot of LASSO regression analysis showing the relationship between gene coefficients and log(λ). The x-axis represents log(λ) (the logarithm of the penalty parameter), and the y-axis represents gene coefficients. The λ value corresponding to the minimum penalty (lambda.min = 0.008) and the λ value at one standard error (lambda.1se = 0.037) are indicated, which were used to select genes associated with the target phenotype. **(B)** Coefficient trajectory plot of LASSO regression. The x-axis represents Log(λ), and the y-axis represents the coefficients, illustrating how the coefficients change with varying λ. The lambda.min and lambda.1se values are annotated. **(C)** Line plot of cross-validation results. The x-axis represents the number of variables, and the y-axis represents accuracy, showing the cross-validation accuracy of the model under different variable numbers. **(D)** Venn diagram illustrating the overlap between SVM_RF_gene and lasso_gene, with numerical values indicating the gene count and proportion. **(E)** Box plots showing the relative expression levels of *FOS*, *IL12A*, and SLC2A3 genes in the DN group versus the Control group from the GSE96804 dataset, demonstrating expression differences between the two groups. *p < 0.05; ***p < 0.001; ****p < 0.0001. **(F)** Box plots displaying the relative expression levels of *FOS*, *IL12A*, and SLC2A3 genes in the DN group compared to the Control group from the GSE104948 dataset, showing differential expression between the two groups. n s, not significant; **p < 0.01; ***p < 0.001. **(G)** GSEA of *FOS*: running enrichment score plot (line graph) and gene rank heatmap. **(H)** GSEA of *IL12A*: running enrichment score plot (line graph) and gene rank heatmap.

LASSO regression identified three feature genes: *FOS*, *SLC9A3*, and *IL12A* (**Figures 3A, B**). SVM–RFE analysis showed that the predictive accuracy in the DN training set was highest when five genes were included (**Figure 3C**). The intersection of results from both methods confirmed *FOS*, *SLC9A3*, and *IL12A* as key signature genes (**Figure 3D**). Boxplot analysis in the training and validation sets revealed that only *FOS* and *IL12A* were consistently and significantly downregulated in the DN group (both *p* < 0.05), with concordant expression trends across datasets; thus, these two genes were selected as core key genes for subsequent analyses (**Figures 3E, F**).

A nomogram prediction model based on *FOS* and *IL12A* was constructed (**Supplementary Figure 2A**). The Hosmer–Lemeshow test (*p* > 0.05) indicated good model fit (**Supplementary Figure 2B**). ROC curve analysis (AUC > 0.7) (**Supplementary Figure 2C**) suggested good predictive performance, and decision curve analysis indicated that the nomogram model has potential clinical utility (**Supplementary Figure 2D**).

GSEA was performed with a significance threshold of *p* < 0.05. *IL12A* was enriched in 20 pathways, with its top 10 enriched pathways being: epithelial–mesenchymal transition (EMT), E2F targets, G2M checkpoint, MYC targets V1, KRAS signaling up, allograft rejection, heme metabolism, mitotic spindle, apical junction, and mTORC1 signaling (**Supplementary Table 16**). *FOS* was enriched in 30 pathways, with its top 10 including: oxidative phosphorylation, fatty acid metabolism, adipogenesis, TNF-α signaling via NF-κB, xenobiotic metabolism, mTORC1 signaling, hypoxia, MYC targets V1, glycolysis, and DNA repair (**Supplementary Table 17**). The top 10 enriched pathways for both genes encompassed processes related to cell cycle regulation, metabolism, and inflammation (**Figures 3G, H**).

### 3.4 Immune infiltration analysis in DN

**Figure 4:**
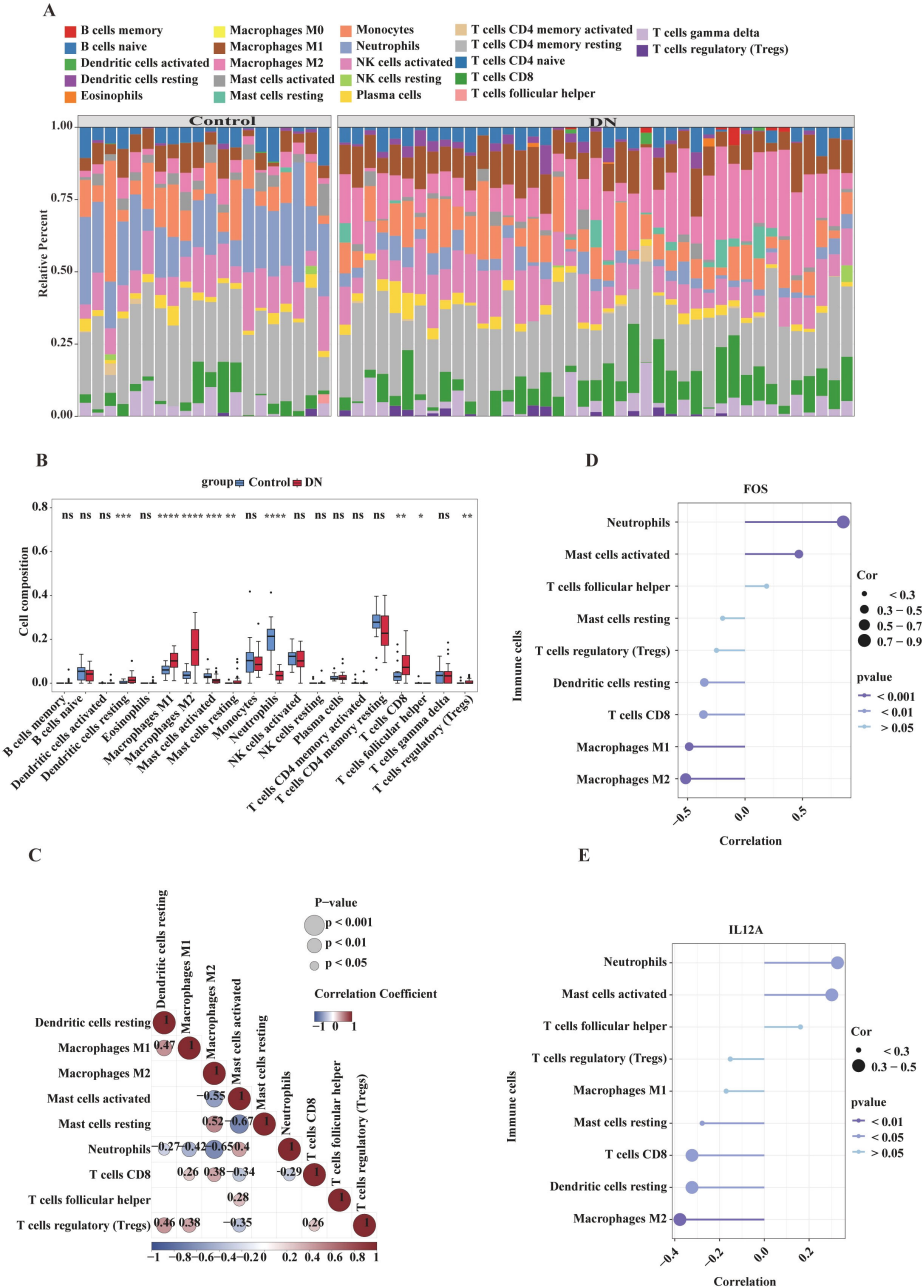
Analysis of immune cell infiltration and its association with key genes in diabetic nephropathy versus control group. **(A)** Stacked heatmap of the relative proportions of immune cells, showing the distribution of various immune cell types in the control group and the DN group. Different colors represent different immune cell types. **(B)** Box plots illustrating the proportions of various immune cell types in the control group versus the DN group. The x-axis represents immune cell types, and the y-axis represents the proportion of cells. Red and blue boxes denote the DN group and control group, respectively, demonstrating differences in immune cell proportions between the two groups. **(C)** Correlation heatmap among immune cells. The color gradient (from blue to red) and annotated numerical values indicate the correlation coefficients between different immune cell types, reflecting the degree of association. The size and color of the points are differentiated based on P values (*p* < 0.001, *p* < 0.01, *p* < 0.05) to indicate significance. **(D)** Scatter plot of the correlation between *FOS* gene expression and various immune cell types. The x-axis represents the correlation coefficient, and the y-axis represents immune cell types. The color and size of the points indicate the P value. **(E)** Scatter plot of the correlation between *IL12A* gene expression and various immune cell types, showing the association of *IL12A* gene expression with immune cell infiltration.

This study evaluated differences in the immune landscape during the progression of DN within the training set. The infiltration levels of 22 immune cell types were scored between patients and normal controls. As shown in **Figure 4A**, resting CD4 memory T cells were the most abundant population in both control and disease samples. To further examine differences in immune cell infiltration between DN and normal samples, violin plots revealed that 9 out of the 22 immune cell types exhibited significantly different infiltration levels (*p* < 0.05) (**Figure 4B**). These differential immune cells included resting dendritic cells, M1 macrophages, M2 macrophages, activated mast cells, resting mast cells, neutrophils, CD8^+^ T cells, T follicular helper cells, and regulatory T cells (Tregs).

Correlation analysis among these differential immune cells (significance threshold: *p* < 0.05 and |cor| > 0.3) revealed the strongest correlation, a negative one, between activated mast cells and resting mast cells (cor = –0.67, *p* < 0.05) (**Figure 4C**; **Supplementary Table 18**). Concurrently, the correlations between *FOS*/*IL12A* expression and these differential immune cells were analyzed. The results indicated that *FOS* expression correlated most strongly with neutrophils (cor = 0.856, *p* < 0.05), while *IL12A* expression showed its strongest negative correlation with M2 macrophages (cor = –0.377, *p* < 0.05) (**Figures 4D, E**; **Supplementary Table 19**). Additionally, to explore the potential regulatory mechanisms of *FOS* and *IL12A*, lncRNA–miRNA–mRNA networks were constructed. As shown in **Supplementary Figure 3A**, 7 miRNAs and 9 lncRNAs were significantly associated with *IL12A*, while 11 miRNAs and 14 lncRNAs were significantly associated with *FOS*.

### 3.5 Identification of key gut microbiota, metabolites, and molecular docking

To further investigate the associations between *FOS*/*IL12A* and gut microbial metabolites, this study retrieved the corresponding relationships linking these key gene targets to specific gut microbiota and metabolites from the gutMGene database. As shown in **Supplementary Figure 3B**, *IL12A* is a predicted target of Faecalibacterium prausnitzii and Akkermansia muciniphila, and is associated with the gut metabolites butyrate and trimethylamine. *FOS* is a predicted target of Lactobacillus acidophilus and is associated with butyrate. Notably, butyrate emerged as a common metabolite target for both key genes.

**Figure 5:**
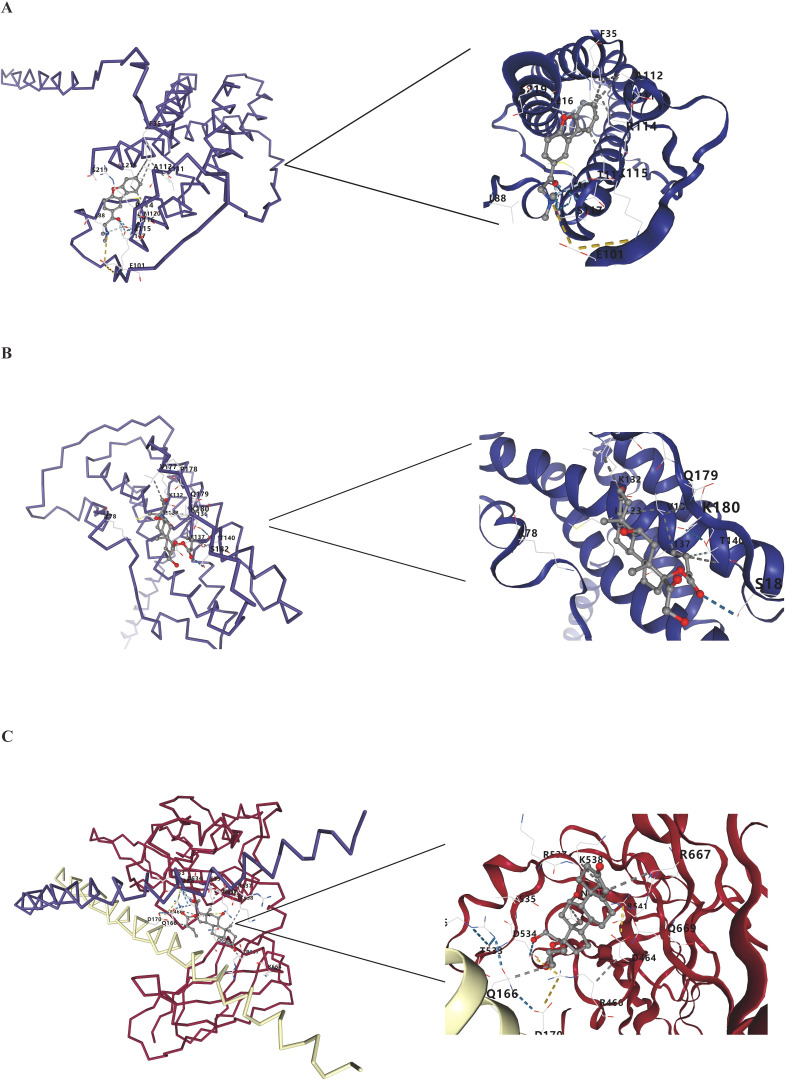
Molecular docking of FOS and IL12A with gut metabolites. **(A)** Molecular docking of *IL12A* with Trimethylamine. **(B)** Molecular docking of *IL12A* with Butyrate. **(C)** Molecular docking of *FOS* with Butyrate.

To investigate the binding potential between the proteins encoded by *FOS*/*IL12A* and these gut metabolites, molecular docking was performed. The results are shown in **Table 3**. Molecular docking results from the CB-Dock2 platform demonstrated stable binding interactions (**Figures 5A–C**), with calculated binding energies of –6.4 kcal/mol for *IL12A* with trimethylamine, –7.0 kcal/mol for *IL12A* with butyrate, and –7.3 kcal/mol for *FOS* with butyrate. SwissDock molecular docking results showed binding energies of *IL12A* with trimethylamine and butyrate at –5.8 kcal/mol and –5.7 kcal/mol, respectively, and the binding energy of *FOS* with butyrate was –5.7 kcal/mol. These findings from both platforms preliminarily validate the potential interactions between the two genes and the gut metabolites.

**Table 3:** Analysis docking across different platforms.

Platform	Mol	gene	Score (kcal/mol)
CB-Dock2	Trimethylamine	IL12	-6.4 kcal/mol
	Butyrate		-7.0 kcal/mol
	Butyrate	FOS	-7.3 kcal/mol
SwissDock	Trimethylamine	IL12	-5.8 kcal/mol
	Butyrate		-5.7 kcal/mol
	Butyrate	FOS	-7.0 kcal/mol

### 3.6 Single-cell analysis of DN

**Figure 6:**
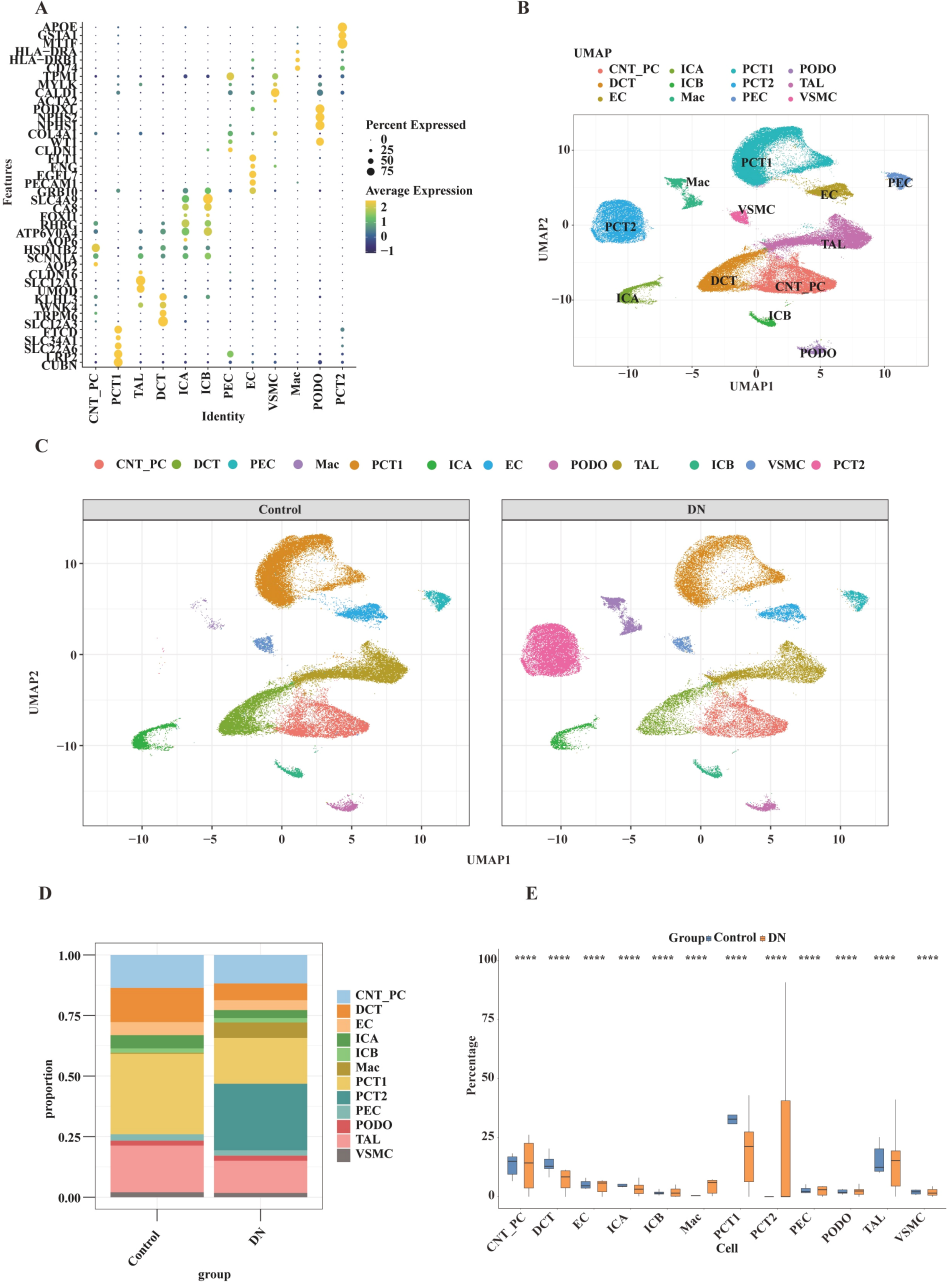
Single-cell transcriptomic analysis of renal cells in DN and control groups. **(A)** Dot plot displaying the expression of signature genes across different renal cell types. The x-axis represents cell identity, and the y-axis shows the signature genes. Dot size indicates the percentage of cells expressing the gene (Percent Expressed), and color represents the average expression level. **(B)** UMAP projection of renal cells, with different colors representing distinct cell types, illustrating the distribution of cells in two-dimensional space. **(C)** UMAP projection comparing renal cells from the control and DN groups. Different colors denote different cell types, highlighting distribution differences between the two groups in UMAP space. **(D)** Stacked bar chart showing the proportional composition of various renal cell types in the control versus DN groups. Different colors represent different cell types, demonstrating differences in cellular composition between groups. **(E)** Box plots comparing the proportions of each renal cell type between the control and DN groups. The x-axis represents cell types, and the y-axis shows the percentage. Differences in cell type proportions between groups are shown, with ****p < 0.0001.

To comprehensively investigate DN at the single-cell level, quality control was performed on the single-cell dataset (**Supplementary Figures 4A, B**). The top 2,000 HVGs were selected and subjected to variance-stabilizing transformation to address heteroscedasticity in downstream analysis (**Supplementary Figure 4C**). Linear dimensionality reduction revealed a decreasing standard deviation with increasing dimensions, with 20 dimensions identified as optimal for cell clustering (**Supplementary Figures 4D, E**). Cell clusters were annotated based on canonical markers and verified through expression patterns (**Figure 6A**; **Supplementary Figures 5A, B**), resulting in the identification of 12 distinct cell types (**Figure 6B**; **Supplementary Figure 5C**).

UMAP visualization revealed clear clustering of cells in the control group, while the spatial distribution of cells in the DN group was significantly altered. This included disorganization and rearrangement of existing cell clusters, as well as the emergence of unique clusters not present in the control group (e.g., the pink cluster) (**Figure 6C**), indicating that DN substantially remodels the cellular landscape. Further analysis of cell type abundance differences between DN and control groups showed that proximal convoluted tubule (PCT) cells exhibited the most significant differences, with PCT1 being the most abundant in controls, while PCT2 became the most abundant in the disease state (**Figure 6D**). Additionally, a boxplot analysis confirmed that all 12 annotated cell types exhibited significant abundance changes in DN compared to controls (**Figure 6E**).

### 3.7 Expression of key genes in annotated cell types

**Figure 7:**
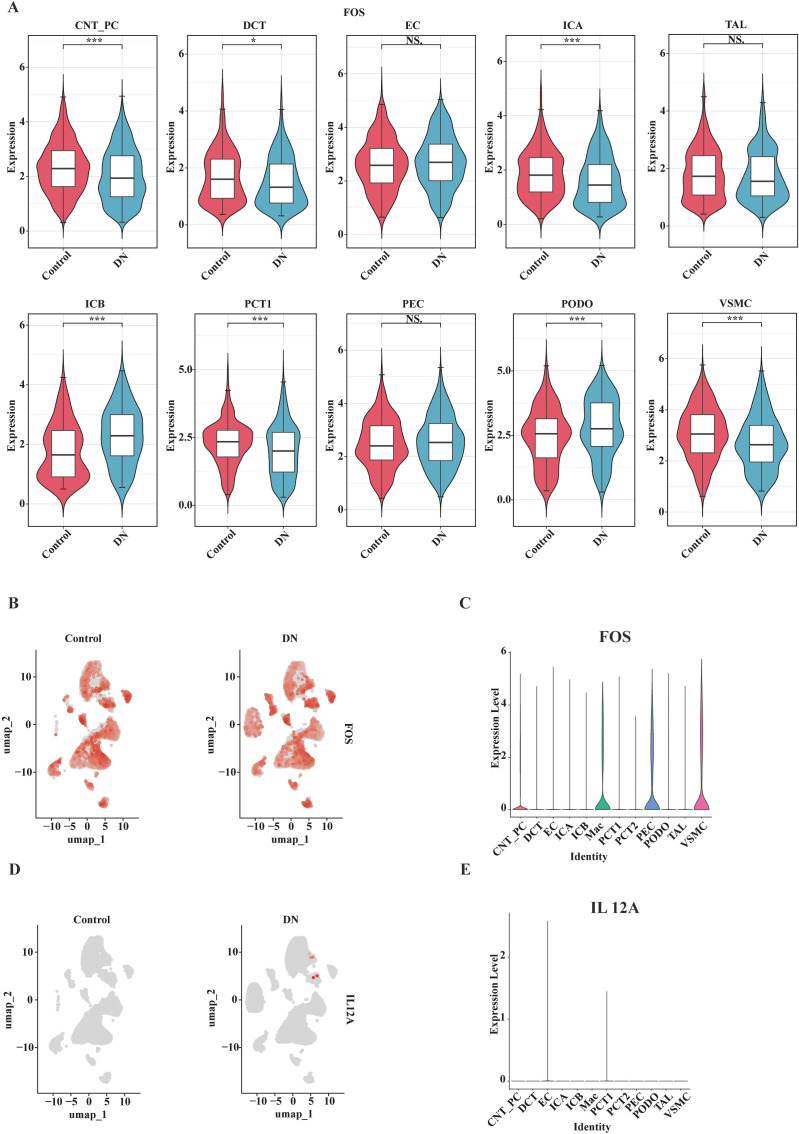
Expression of key genes in renal cells of DN and control groups. **(A)** Violin plots comparing gene expression between the control (red) and DN (blue) groups across different renal cell types (e.g., CNT_PC, DCT, PCT1). The plots illustrate differences in expression distribution across cell types and between groups. ns, not significant; *p < 0.05; ***p < 0.001. **(B)** UMAP projection of *FOS* expression in the control and DN groups. Red dots represent cells expressing *FOS*, demonstrating the spatial expression pattern of *FOS* between the two groups. **(C)** Violin plot showing the expression of *FOS* across different renal cell types, highlighting cell type-specific expression differences. **(D)** UMAP projection of *IL12A* expression in the control and DN groups, illustrating the spatial distribution pattern of *IL12A* between groups. **(E)** Violin plot displaying the expression of *IL12A* across different renal cell types, demonstrating cell type-specific expression variations.

To identify the key cell populations, the expression and distribution of the key genes *FOS* and *IL12A* across all annotated cell types were examined. Violin plots demonstrated that, compared to the control group, FOS expression was significantly altered in DN across several cell types, including PCT Cell 1 (PCT1), Distal Convoluted Tubule cell (DCT), Connecting Tubule and Principal Cell (CNT_PC), Type A Intercalated Cell (ICA), Type B Intercalated Cell (ICB), Podocyte (PODO), and Renal Vascular Smooth Muscle Cell (VSMC) (**Figure 7A**). UMAP visualization further revealed that *FOS* was widely distributed and expressed across multiple cell types (**Figures 7B, C**). In contrast, *IL12A* expression was restricted to PCT1 and Endothelial Cells (ECs), with lower overall abundance (**Figures 7D, E**).

Although *FOS* showed differential expression in PCT1, its expression in ECs was not significantly altered. Since both *FOS* and *IL12A* exhibited significant differential expression specifically within PCT1, PCT1 was preliminarily selected as the key cell type for further focused analysis.

### 3.8 Pseudotime trajectory analysis of key cell clusters

**Figure 8:**
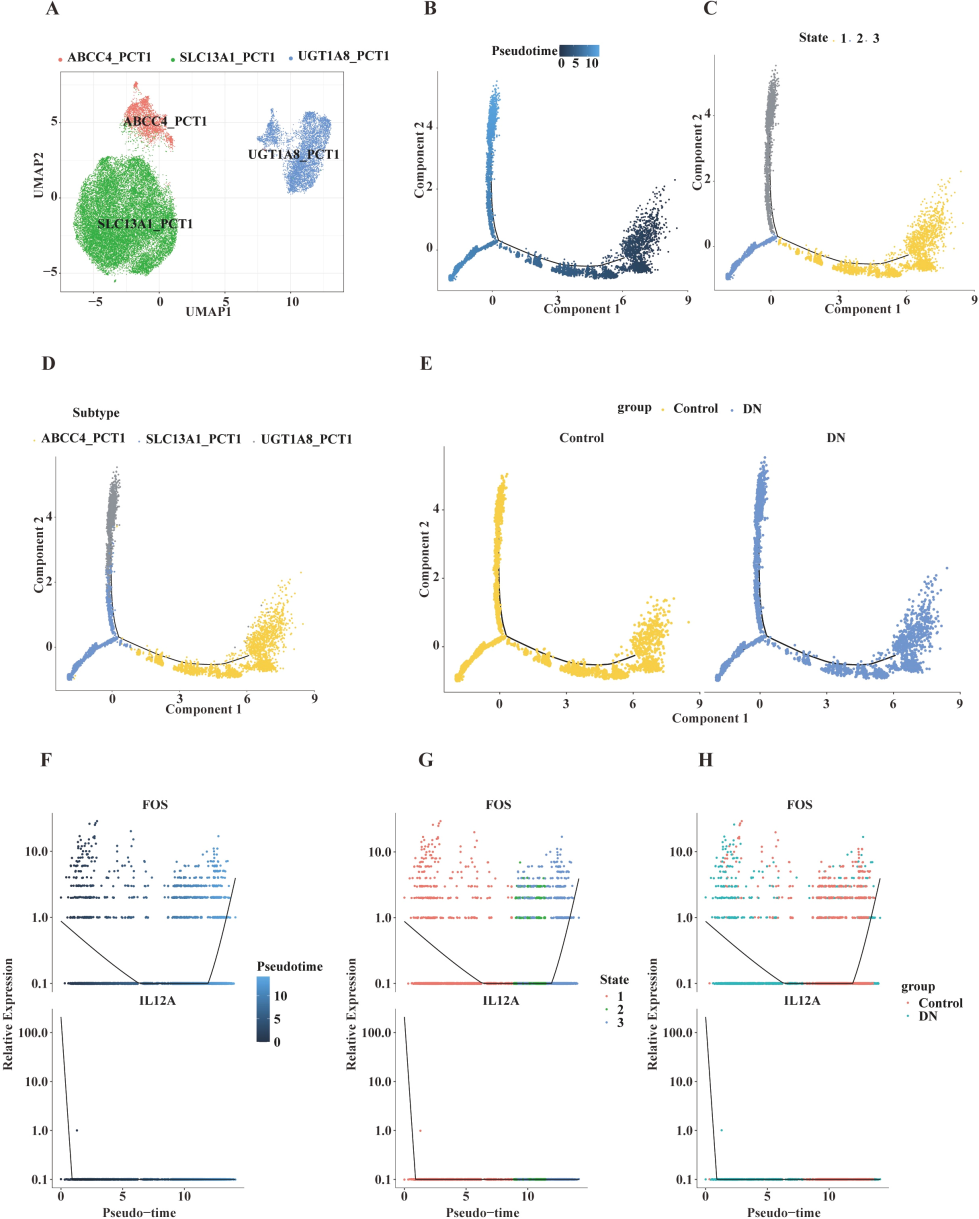
Subtype analysis of renal proximal convoluted tubule cells (PCT1) and pseudotemporal expression dynamics of key genes. **(A)** UMAP projection of PCT1 subtypes, with different colors representing distinct subtypes (ABCC4_PCT1, SLC3A1_PCT1, UGT1A8_PCT1), illustrating the distribution of subtypes in two-dimensional space. **(B)** Trajectory plot of pseudotime analysis. The x-axis represents Component 1, and the y-axis represents Component 2. Blue dots with a color gradient indicate pseudotime progression (0–10), showing cellular transitions along pseudotime. **(C)** Pseudotime trajectory plot colored by different states (State 1, 2, 3), demonstrating the distribution of cells across states along pseudotime. **(D)** Pseudotime trajectory plot colored by PCT1 subtypes (ABCC4_PCT1, SLC3A1_PCT1, UGT1A8_PCT1), showing subtype-specific progression along pseudotime. **(E)** Pseudotime trajectory plot comparing control and DN groups, with different colors representing each group, highlighting differential cellular transitions along pseudotime. **(F)** Scatter plots showing the relative expression changes of *FOS* and *IL12A* along pseudotime. The plots integrate pseudotime progression and gene expression levels, with a blue color gradient representing pseudotime, illustrating the relationship between gene expression and pseudotemporal progression. **(G)** Scatter plots displaying the relative expression changes of *FOS* and *IL12A* along pseudotime across different cellular states (State 1, 2, 3). Different colors represent different states, demonstrating the relationship between gene expression, cellular states, and pseudotime. **(H)** Scatter plots showing the relative expression changes of *FOS* and *IL12A* along pseudotime in control versus DN groups. Different colors represent each group, revealing differential gene expression patterns between groups along pseudotime.

To explore the heterogeneity of PCT1, UMAP clustering was performed, subdividing them into three distinct subclusters (**Figure 8A**; **Supplementary Figures 6A, B**). Based on marker gene expression, these subclusters were annotated as UGT1A8_PCT1, SLC13A1_PCT1, and ABCC4_PCT1 subtypes. In the DN group, the distribution of SLC13A1_PCT1 appeared more scattered, the overlap between ABCC4_PCT1 and SLC13A1_PCT1 was reduced, and the spatial distribution and density of UGT1A8_PCT1 were altered (**Supplementary Figure 6C**), suggesting that DN modulates the composition and cellular states of the PCT1 subpopulations.

GSEA (using the h.all.v2023.2.Hs.symbols.gmt gene set) revealed distinct pathway activities across the subclusters (**Supplementary Figures 6D–F**). UGT1A8_PCT1 showed activation of the EMT pathway. SLC13A1_PCT1 exhibited activation of the IL-6–JAK–STAT3 signaling pathway, involved in inflammatory adaptive responses (37). ABCC4_PCT1 displayed activation of the TNF-α–NF-κB signaling pathway, which mediates the coordinated regulation of inflammation and substance transport (38).

Trajectory analysis of PCT1 indicated a left-to-right progression of differentiation (darker blue indicating earlier differentiation) (**Figure 8B**). The ABCC4_PCT1, SLC13A1_PCT1, and UGT1A8_PCT1 subclusters predominantly emerged at differentiation stages 1, 0, and 2, respectively (**Figures 8C, D**). Cells from both the control and DN groups showed distinct distributions along Components 1 and 2 (**Figure 8E**), confirming that DN reshapes cellular phenotypic features. Expression analysis of key genes along the trajectory revealed that *FOS* expression initially decreased, then increased during differentiation, while *IL12A* expression progressively declined and was silenced in later stages (**Figures 8F–H**).

### 3.9 Communication analysis of key cell clusters

**Figure 9:**
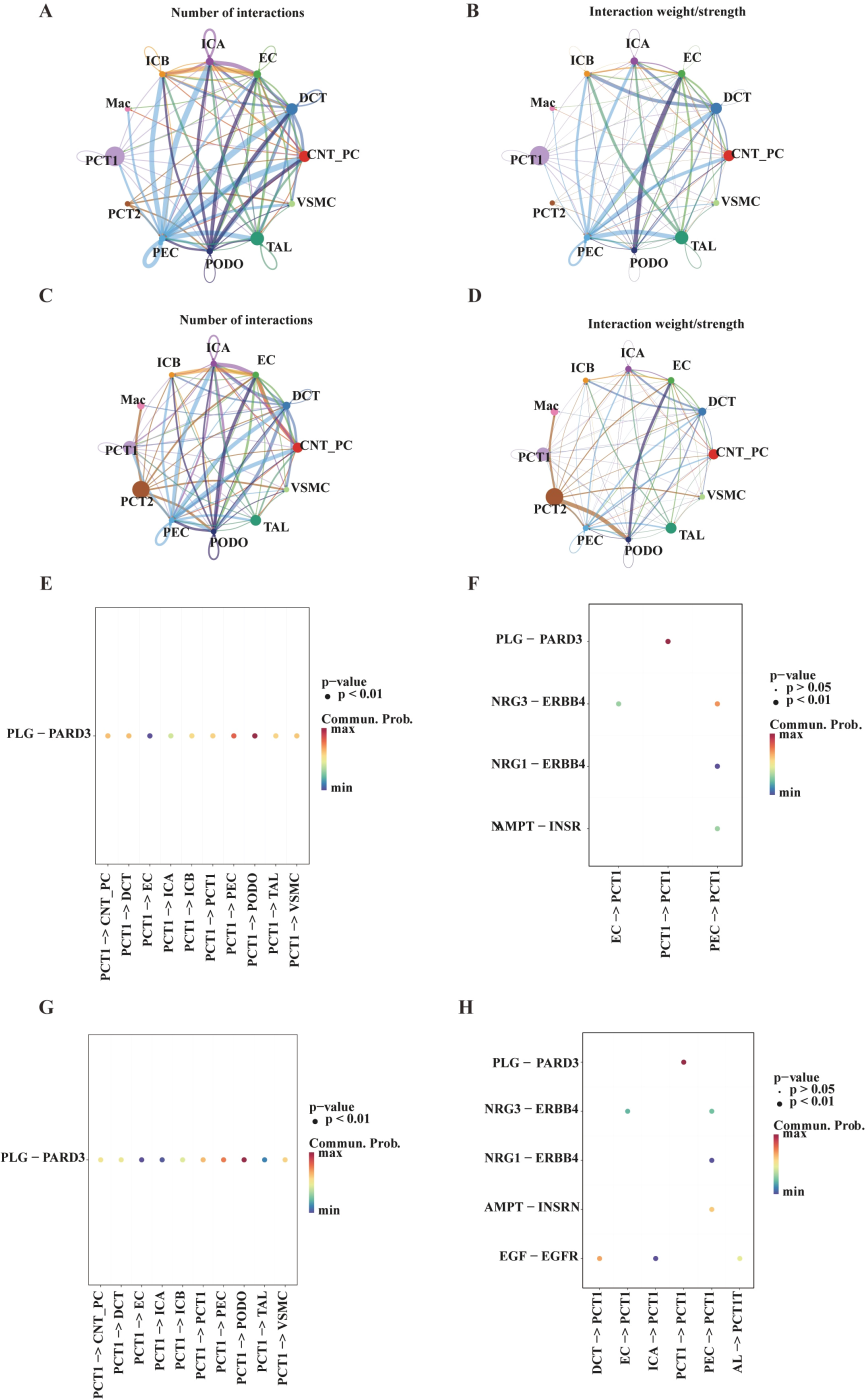
Analysis of intercellular interactions and key ligand-receptor pairs among renal cell types. **(A)** Network diagram illustrating the number of interactions between different cell types (e.g., ICB, ICA, EC) in the control group. Lines and nodes represent connectivity between cells, reflecting the distribution of interaction counts among cell types. **(B)** Network diagram depicting the strength of interactions between different cell types in the control group. Line color and thickness represent the weight/intensity of interactions, demonstrating variations in interaction strength between cell types. **(C)** Network diagram showing the number of cellular interactions in the diabetic nephropathy (DN) group, presenting the association of interaction counts among these cells. **(D)** Network diagram displaying the strength of interactions between cell types in the DN group, illustrating the distribution of interaction intensities. **(E)** Dot plot analyzing the PLG-PARD3 ligand-receptor pair across cell type pairs in the control group. The x-axis represents cell type pairs, and the y-axis shows relevant metrics. The color gradient represents the maximum and minimum of “Common.Prob.”, with p value annotated (*p* < 0.01). **(F)** Dot plot analyzing PLG-PARD3, NRG3-ERBB4, and NAMPT-INSR ligand-receptor pairs in specific cell type pairs of the control group. The color gradient represents the maximum and minimum of “Common.Prob.”, with P values annotated (*p* < 0.05, *p* < 0.01). **(G)** Dot plot analyzing the PLG-PARD3 ligand-receptor pair across different cell type pairs in the DN group. The x-axis represents cell type pairs, and the y-axis shows relevant metrics. The color gradient represents the maximum and minimum of “Common.Prob.”, with P value annotated (*p* < 0.01). **(H)** Dot plot analyzing PLG-PARD3, NRG3-ERBB4, NAMPT-INSR, and EGF-EGFR ligand-receptor pairs in specific cell type pairs (e.g., DCT→PCT1) of the DN group. The color gradient represents the maximum and minimum of “Common.Prob.”, with P values annotated (*p* < 0.05, *p* < 0.01).

To examine the interaction patterns between the key cell cluster (PCT1) and other annotated cell populations, cell–cell communication analysis was performed. As shown in **Figures 9A–D**, the DN group exhibited a significant decrease in both the number and strength of interactions involving PCT1 compared to the Control group, while interactions involving PCT2 showed a marked increase.

Further analysis identified the ligand–receptor pairs with the highest communication probabilities. In the Control group, when PCT1 acted as a signal-sending cell (source), the highest probability interaction was PCT1 sending the PLG ligand to bind the PARD3 receptor on PODO cells. When PCT1 acted as a signal-receiving cell (target), the highest probability interaction was PEC sending the NRG3 ligand to bind the ERBB4 receptor on PCT1 (**Figures 9E, F**). In the DN group, the highest probability interaction for PCT1 as a source remained the same: sending PLG to PODO’s PARD3. However, when PCT1 acted as a target, the highest probability interaction shifted to DCT sending the EGF ligand to bind the EGFR receptor on PCT1 (**Figures 9G, H**).

These results indicate that the DN state significantly remodels the cellular communication network in renal tissue. The communication activity of PCT1 is attenuated, while PCT2 exhibits enhanced communication. Notably, while the core ligand–receptor pattern for PCT1 as a signal receiver undergoes disease-specific alterations, its key outgoing communication pathway with PODO remains stable. These changes in cellular communication are likely critical in regulating the pathophysiology of DN.

### 3.10 Significant downregulation of *FOS* and *IL12A* in the blood of patients with diabetic nephropathy

**Figure 10:**
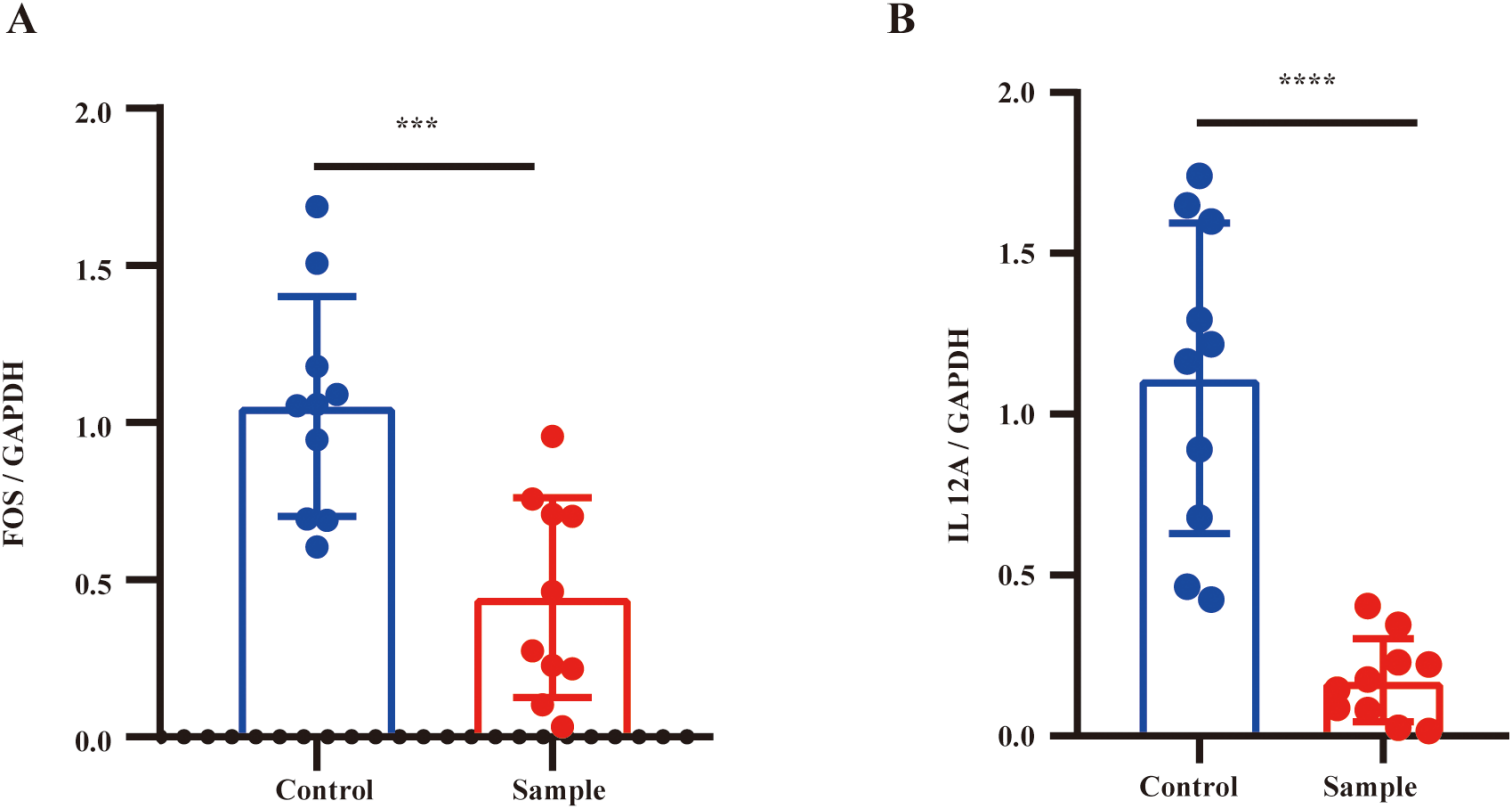
Expression changes of FOS and IL12A in diabetic nephropathy samples. **(A)** Relative expression level of *FOS* in control group and diabetic nephropathy sample group (normalized to GAPDH). **(B)** Relative expression level of *IL12A* in control group and diabetic nephropathy sample group (normalized to GAPDH). n = 10, with three biological replicates. Statistical significance: ***p < 0.001, ****p < 0.0001.

Various inflammatory and renal injury biomarkers in serum and urine are significantly associated with the development and progression of DN (39). Additionally, it has been suggested that expression levels in peripheral blood can reflect the local pathological state of the kidney (40). To further validate the expression patterns of the identified key genes in clinical samples, RT-qPCR was performed on blood samples from patients with DN and healthy controls. Consistent with the transcriptomic analysis results, mRNA expression levels of both *FOS* and *IL12A* were significantly downregulated in the blood samples of patients with DN compared to healthy controls (*p* < 0.05) (**Figures 10A, B**). These results suggest that FOS and IL12A may potentially influence the pathological state of DN in the blood.

## 4 Discussion

This study innovatively integrated druggable genome screening with MR causal inference. It is the first to preliminarily validate that FOS and IL12A may have a potential causal relationship with DN, suggesting that they may serve as druggable targets. Moreover, this study is the first to systematically establish a potential causal and molecular interaction network between key druggable targets and the gut microbiota–metabolite axis. Specifically, *FOS* was preliminarily validated to be associated with Lactobacillus acidophilus, while *IL12A* was linked to beneficial microbes such as Faecalibacterium prausnitzii and Akkermansia muciniphila. Both genes demonstrated stable binding with butyrate, a core gut metabolite, exhibiting binding energies of –7.3 kcal/mol and –7.0 kcal/mol, respectively. *IL12A* also exhibited specific binding with trimethylamine (binding energy: –6.4 kcal/mol). These findings reveal a potential synergistic regulatory mechanism involving the “gut microbiota–metabolite–host druggable gene” axis and highlight direct molecular interactions between gut microbes and host target genes. This paves the way for exploring the functions of target genes and exerting renal protective effects by modulating gut microbiota or supplementing specific metabolites. These insights could provide a theoretical and experimental foundation for future gut–kidney axis-targeted interventions in DN.

*FOS*, a member of the AP-1 transcription factor family, plays a role in regulating cellular stress and inflammatory responses in kidney injury (41, 42). However, its causal role and underlying mechanisms in DN were not well understood. Our study provides the first evidence of FOS’s potential causal association with DN, specifically implicating its central role in the mTORC1 signaling pathway. Downregulation of *FOS* may reduce the transcriptional activation of TSC2—a key negative regulator of mTORC1—thereby relieving the inhibition on mTORC1 signaling. This hypothesis is supported by existing evidence showing that *FOS*, in collaboration with Jun, binds to the AP-1 site in the TSC2 promoter to promote its expression (43). It is well-established that loss of TSC2 function leads to sustained activation of Rheb-GTP, which strongly activates mTORC1 kinase activity (44). This mechanism may synergize with the AGEs/RAGE–PI3K–Akt pathway, which is commonly activated under hyperglycemic conditions. Specifically, AGE binding to RAGE activates PI3K/Akt, and subsequent Akt phosphorylation inhibits TSC2 function (45, 46). These insights suggest a novel mechanism by which *FOS* downregulation may contributes to the pathogenesis of renal disease.

Regarding *IL12A*, our findings challenge its traditional view as solely a pro-inflammatory factor. As a key cytokine regulating Th1-type immune responses, its role in DN remains controversial. While some studies suggest *IL12A* promotes inflammation and exacerbates renal injury (47, 48), our data show significant downregulation of *IL12A* in DN and a negative correlation with M2 macrophages. This apparent contradiction likely arises from the complexity of the immune microenvironment: the functional attenuation of M2 macrophages—commonly regarded as anti-inflammatory and pro-repair cells—coincides with *IL12A* downregulation. This suggests that *IL12A* may play a role in renal tissue repair by modulating the balance of macrophage polarization. Previous studies have shown that *IL12A* can activate the Stat4 signaling pathway, promoting IFN-γ secretion from Th1 cells (49). Zsuzsanna S. Nagy’s team demonstrated that IFN-γ suppresses PPARγ expression in macrophages (50), a master transcription factor essential for M2 polarization. Reduced PPARγ expression leads to diminished levels of characteristic M2 markers, such as Arg1 and CD206 (51, 52). These findings imply a potential regulatory network involving IL12A, IFN-γ, and PPARγ in macrophage polarization in DN, suggesting that IL12A may exert a therapeutic role through this pathway.

The previously reported pro-inflammatory role of *IL12A* might stem from its elevated expression in the highly inflammatory microenvironment of late-stage DN (53, 54). In contrast, the downregulation observed in our study at early stages may reflect functional heterogeneity across disease phases. It is thus hypothesized that in early diabetic nephropathy (DN), reduced *IL12A* levels may lead to insufficient M2 polarization, thereby weakening tissue repair capacity. In later stages, however, the inflammatory storm may trigger compensatory upregulation of *IL12A*, shifting its function toward a pro-inflammatory role. This temporal dynamic could offer a reasonable explanation for the controversies surrounding *IL12A’s* role in DN.

The “gut–kidney axis” is a central focus in DN pathogenesis research. Our study is the first to establish a direct link between key druggable targets and the gut microbiota–metabolite axis: the stable binding of *FOS* and *IL12A* with butyrate (binding energy ≤ –7.0 kcal/mol) and their associations with Lactobacillus acidophilus and Faecalibacterium prausnitzii reveal a novel “microbiota–metabolite–gene” interaction mechanism. Butyrate, a core beneficial metabolite produced by gut microbiota, is closely linked to impaired gut barrier integrity and renal inflammation in DN (55). Our finding that butyrate binds to *FOS*/*IL12A* suggests that exogenous supplementation of butyrate or modulation of relevant microbiota could exert renal protective effects by activating these target proteins. This provides preliminary molecular evidence for “microbiota–metabolite–host gene” interactions and uncovers a new mechanism by which the gut microbiota–metabolite axis contributes to DN pathogenesis.

At the single-cell level, this study preliminarily identified PCT1 as the key effector cell through which FOS and IL12A exert their functions. The observed attenuation of its communication activity and aberrant differentiation trajectory serve as critical features of renal tubular injury in DN. As the primary unit for renal reabsorption and metabolism, PCT dysfunction occurs early in DN (16). Further analysis revealed that the UGT1A8_PCT1 subcluster activated the EMT pathway. UGT1A8, highly expressed in this subcluster and acting as a phase II metabolic enzyme, catalyzes the glucuronic acid conjugation of bilirubin, enhancing its water solubility. Bilirubin and its glucuronide metabolites can scavenge reactive oxygen species (ROS) and inhibit lipid peroxidation, exerting protective effects against oxidative stress (56, 57). Therefore, the activation of the EMT pathway in PCT1 under oxidative stress may be a compensatory response, whereby upregulation of UGT1A8-mediated bilirubin metabolism enhances antioxidant capacity (58). In the present study, the SLC13A1_PCT1 subcluster activated the IL6–JAK–STAT3 signaling pathway. SLC13A1, a sodium–citrate cotransporter, is involved in renal tubular reabsorption, and its dysregulation can impair this process (59). However, this key cell type, identified solely through computational predictions, remains a theoretical concept and requires further experimental validation.

The genetically validated causal targets FOS and IL12A identified in this study could provide novel directions for diabetes drug development. The prediction model constructed based on these two targets demonstrates strong potential for disease risk assessment. Additionally, the identified “gene–microbiome–metabolite” interaction network offers a theoretical foundation for microecological intervention strategies. However, this study is preliminary and exploratory, relying on bioinformatics approaches, and has certain limitations: First, the validation set has a small sample size, and the Mendelian analysis was limited to European populations with statistical limitations, these factors may restrict the generalizability of the results. Furthermore, the causal relationships and mechanisms between FOS, IL12A, and DN require additional verification. Second, the expression of FOS and IL12A was only validated in blood, which may not fully reflect changes in kidney tissue. Future research will aim to expand the sample size, include multi-center populations, and verify the expression of FOS and IL12A using kidney biopsy samples (immunohistochemistry, *in situ* hybridization, Western blot, RT-qPCR). Additionally, methods such as Co-immunoprecipitation (Co-IP) or surface plasmon resonance will be used to validate the molecular binding mechanisms. A DN mouse model with targeted PCT1 intervention will be established to assess renal pathology and urine protein levels. Finally, the nomogram model will be validated in large-scale prospective cohorts to enhance the reliability and clinical applicability of the conclusions. These findings hold significant translational value. FOS and IL12A, as genetically validated causal targets, provide new directions for DN drug development. The predictive model built upon them demonstrates strong disease risk assessment capability. Furthermore, the identified “gene-microbiota-metabolite” interaction network offers a theoretical basis for microecological intervention strategies.

## 5 Conclusion

This study systematically explored potential therapeutic targets and pathogenic mechanisms of DN, yielding several key findings: First, *FOS* and *IL12A* were identified as central therapeutic targets, both showing significantly reduced expression in DN. A logistic regression model constructed based on these targets demonstrated excellent predictive performance (AUC > 0.7), highlighting them as promising candidates for precision treatment of DN. Both genes were co-enriched in the mTORC1 signaling pathway and exhibited significant associations with key pathological processes of DN, such as oxidative phosphorylation and EMT. Additionally, *FOS* and *IL12A* contribute to the remodeling of the renal immune microenvironment. Analysis of the gut microbiota–metabolite axis revealed preliminary regulatory associations between Lactobacillus/*FOS*/butyrate and Proteus/*IL12A*/butyrate, with molecular docking experiments suggesting stable binding interactions with butyrate and trimethylamine. Single-cell sequencing analysis identified PCT1 as the primary cell type with differential expression of these targets. These findings propose that *FOS* and *IL12A* may play regulatory roles in the cross-regulation of pathways and gut–kidney axis interactions, though specific mechanisms require further experimental validation. This study provides a preliminary theoretical foundation for the experimental investigation of precision treatment targets in DN.

## Data Availability

Requests to access the datasets should be directed to https://www.ncbi.nlm.nih.gov/geo/.

## Glossary

DN: Diabetic Nephropathy
DGGs: Druggable Genes
MR: Mendelian Randomization
RT-qPCR: Reverse Transcription-quantitative Polymerase Chain Reaction
GSEA: Gene Set Enrichment Analysis
scRNA-seq: single-cell RNA sequencing
T2DM: Type 2 Diabetes Mellitus
ESRD: End-Stage Renal Disease
IVs: Instrumental Variables
SNPs: Single nucleotide Polymorphisms
DEGs: Differentially Expressed Genes
eQTL: Expression Quantitative Trait Loci
GWAS: Genome-wide Association Study
ORs: Odds Ratios
GO: Gene Ontology
KEGG: Kyoto Encyclopedia of Genes and Genomes
ceRNA: Competing Endogenous RNA
lncRNA: Long Non-coding RNA
miRNA: MicroRNA
mRNA: Messenger RNA
SCFAs: Short-chain fatty acids
TMAO: Trimethylamine N-oxide
IVW: Inverse-Variance Weighted
LASSO: Least Absolute Shrinkage and Selection Operator
SVM-RFE: Support Vector Machine-Recursive Feature Elimination
AUC: Area Under the Curve
mTORC1: Mammalian Target of Rapamycin Complex 1
PI3K-Akt: Phosphatidylinositol 3-kinase–Protein Kinase B
HIF-1: Hypoxia-inducible Factor 1
UMAP: Uniform Manifold Approximation and Projection
PCT: Proximal Convoluted Tubule
DCT: Distal Convoluted Tubule
CNT_PC: Connecting Tubule and Principal Cell
ICA: Type A Intercalated Cell
ICB: Type B Intercalated Cell
PODO: Podocyte
VSMC: Renal Vascular Smooth Muscle Cell
ECs: Endothelial Cells
EMT: Epithelial-mesenchymal Transition
JAK-STAT3: Janus Kinase–Signal Transducer and Activator of Transcription 3
TNFA-NFκB: Tumor Necrosis Factor Alpha–Nuclear Factor Kappa B
PEC: Peritubular Capillary Endothelial Cell
EGF: Epidermal Growth Factor
EGFR: Epidermal Growth Factor Receptor
PLG: Plasminogen
PARD3: Partitioning Defective 3
NRG3: Neuregulin 3
ERBB4: V-Erb-B2 Avian Erythroblastic Leukemia Viral Oncogene Homolog 4
AP-1: Activator Protein 1
TSC2: Tuberous Sclerosis Complex 2
AGEs: Advanced Glycation End Products
RAGE: Receptor for Advanced Glycation End Products
IFN-γ: Interferon Gamma
PPARγ: Peroxisome Proliferator-Activated Receptor Gamma
ROS: Reactive Oxygen Species
AUCcell: Area Under the Curve for Cellular Activity
CRGs: Complement Related Genes
FRGs: Ferroptosis Related Genes
BP: Biological Process
CC: Cellular Component
MF: Molecular Function
GEO: Gene Expression Omnibus
CIBERSORT: Cell-type Identification By Estimating Relative Subsets of RNA Transcripts
SD: Standard Deviation
TNF-α: Tumor Necrosis Factor Alpha
NF-κB: Nuclear Factor Kappa B
JAK: Janus Kinase
STAT3: Signal Transducer and Activator of Transcription 3
Arg1: Arginase 1
CD206: Cluster of Differentiation 206
Ct: Cycle Threshold
GAPDH: Glyceraldehyde-3-Phosphate Dehydrogenase
PCR: Polymerase Chain Reaction
