# Supplementary figures and images for: Therapeutic targets for diabetic nephropathy identified by druggable genome mendelian randomization: the role of the gut microbiota-metabolite axis
Source: Front Endocrinol (Lausanne). 2026 May 8;17:1817400. doi: 10.3389/fendo.2026.1817400 (PMC13193880; doi:10.3389/fendo.2026.1817400)

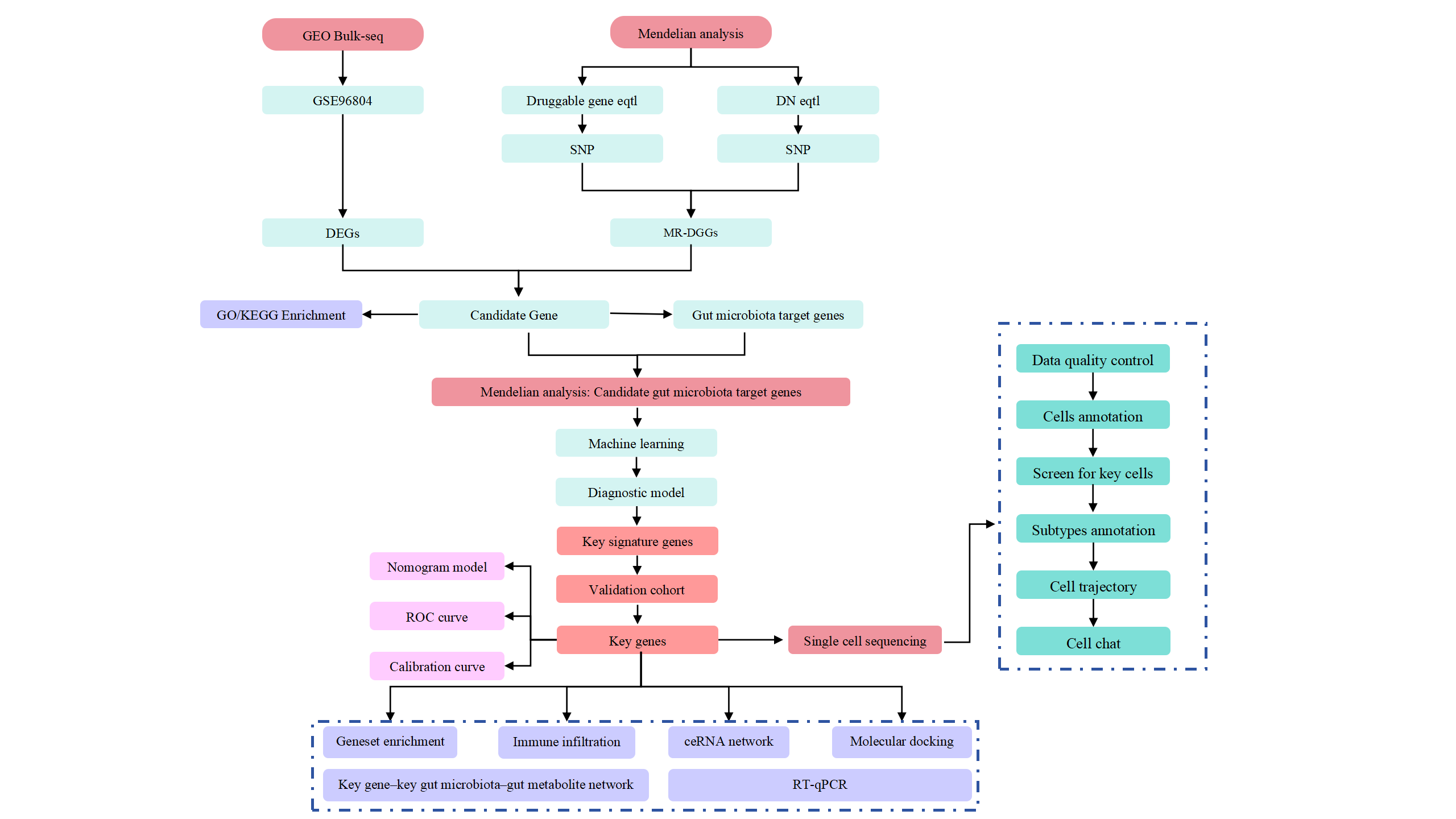

Supplement: Supplementary file 1 [file Image1.tif]

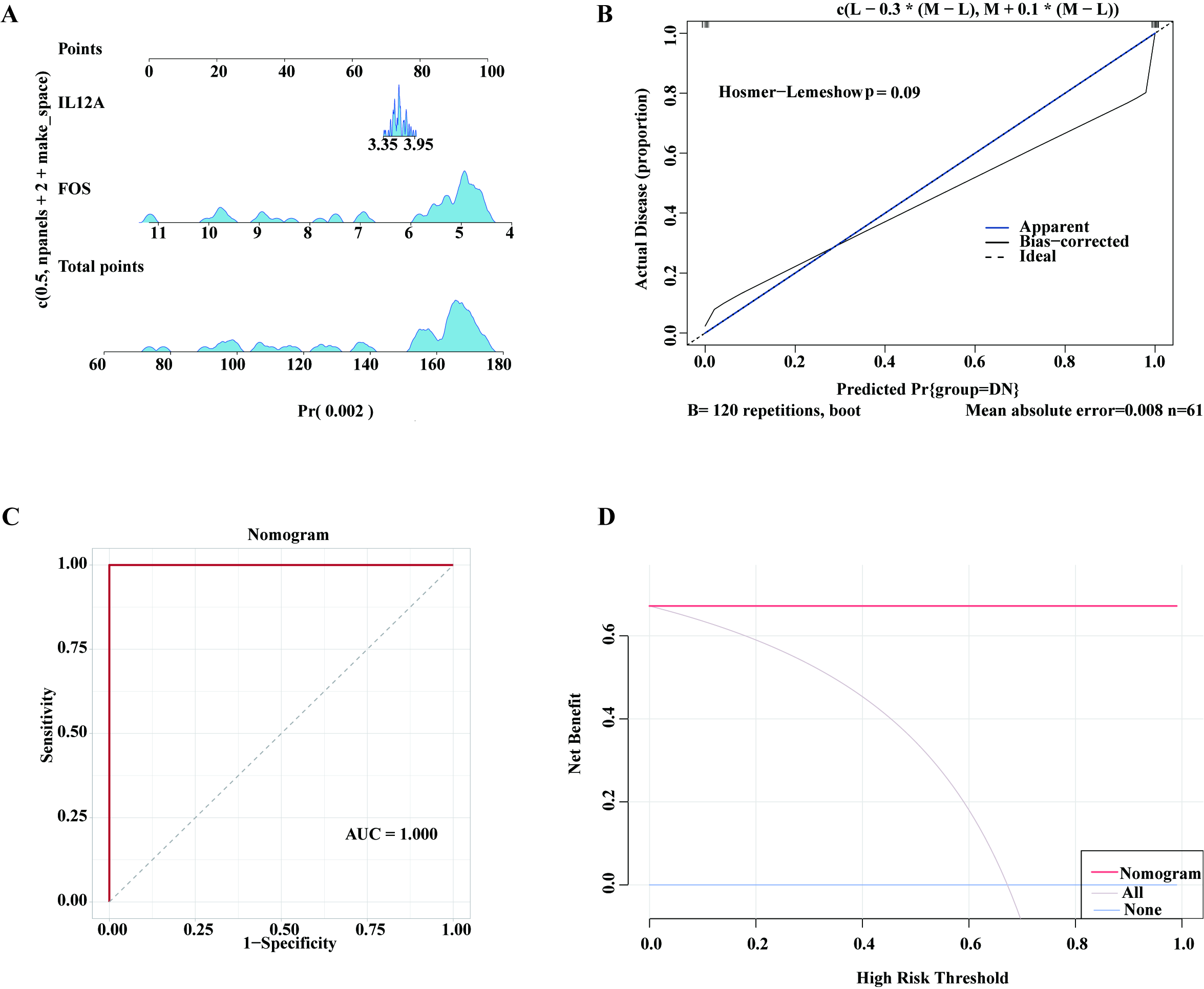

Supplement: Supplementary file 2 [file Image2.tif]

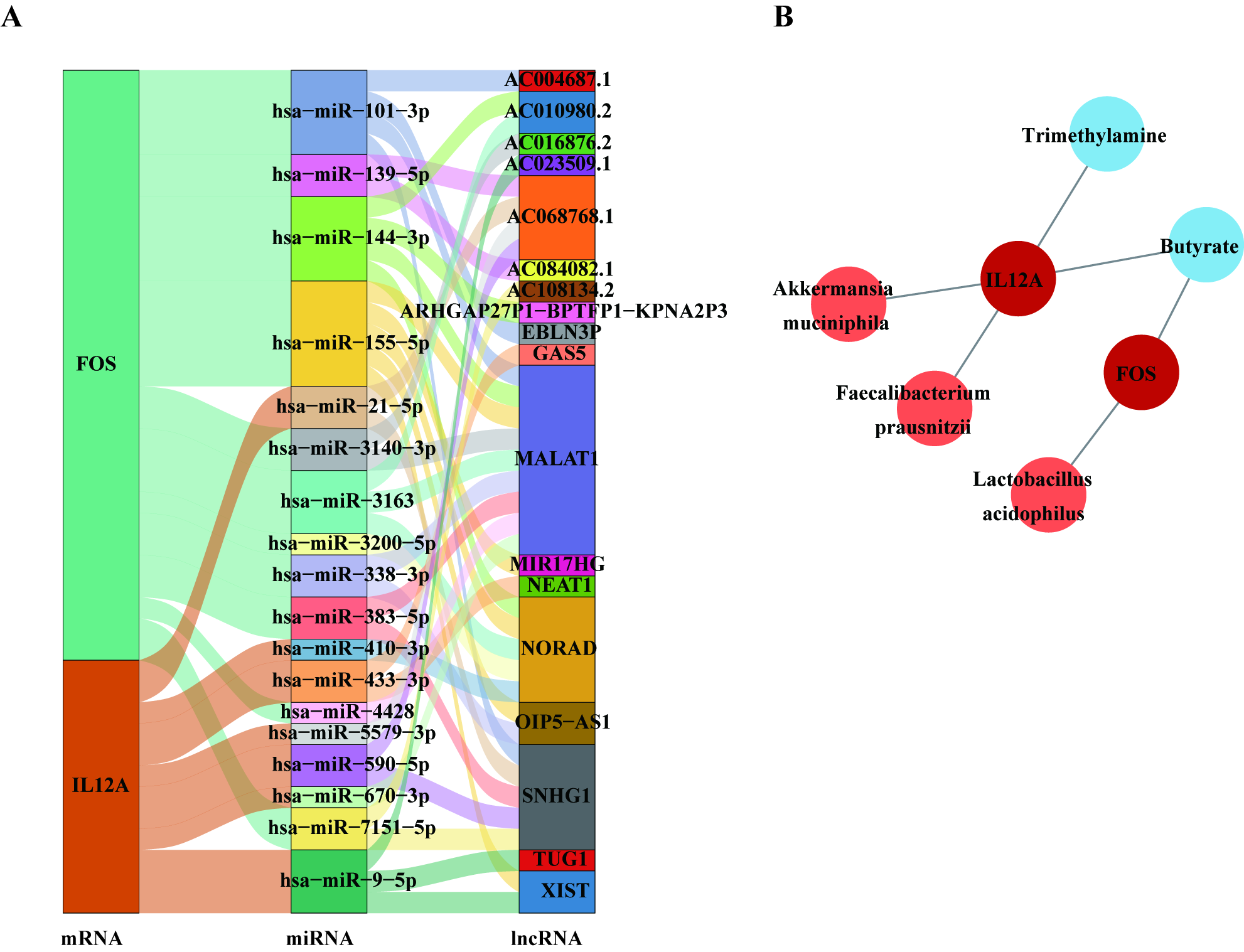

Supplement: Supplementary file 3 [file Image3.tif]

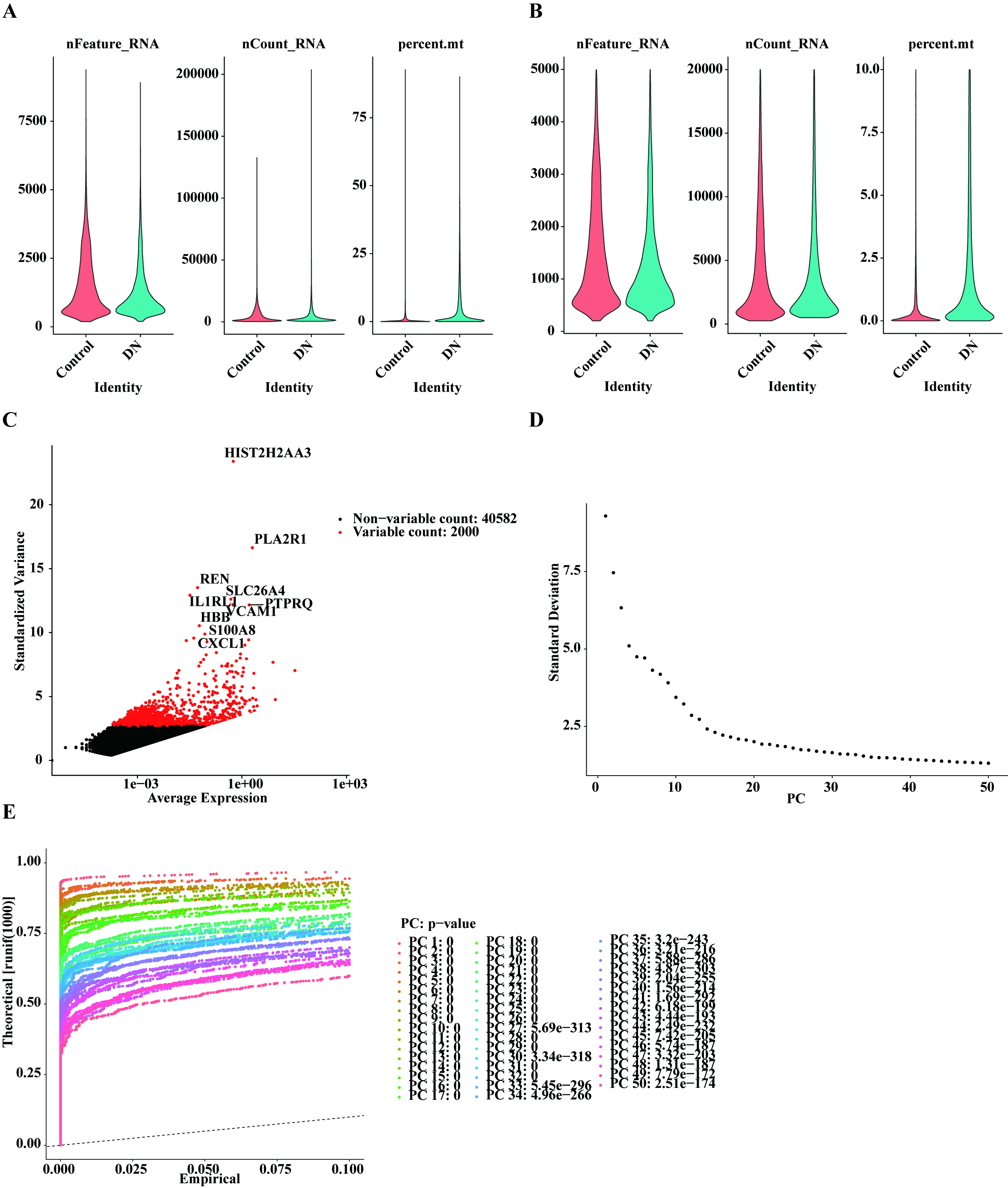

Supplement: Supplementary file 4 [file Image4.tif]

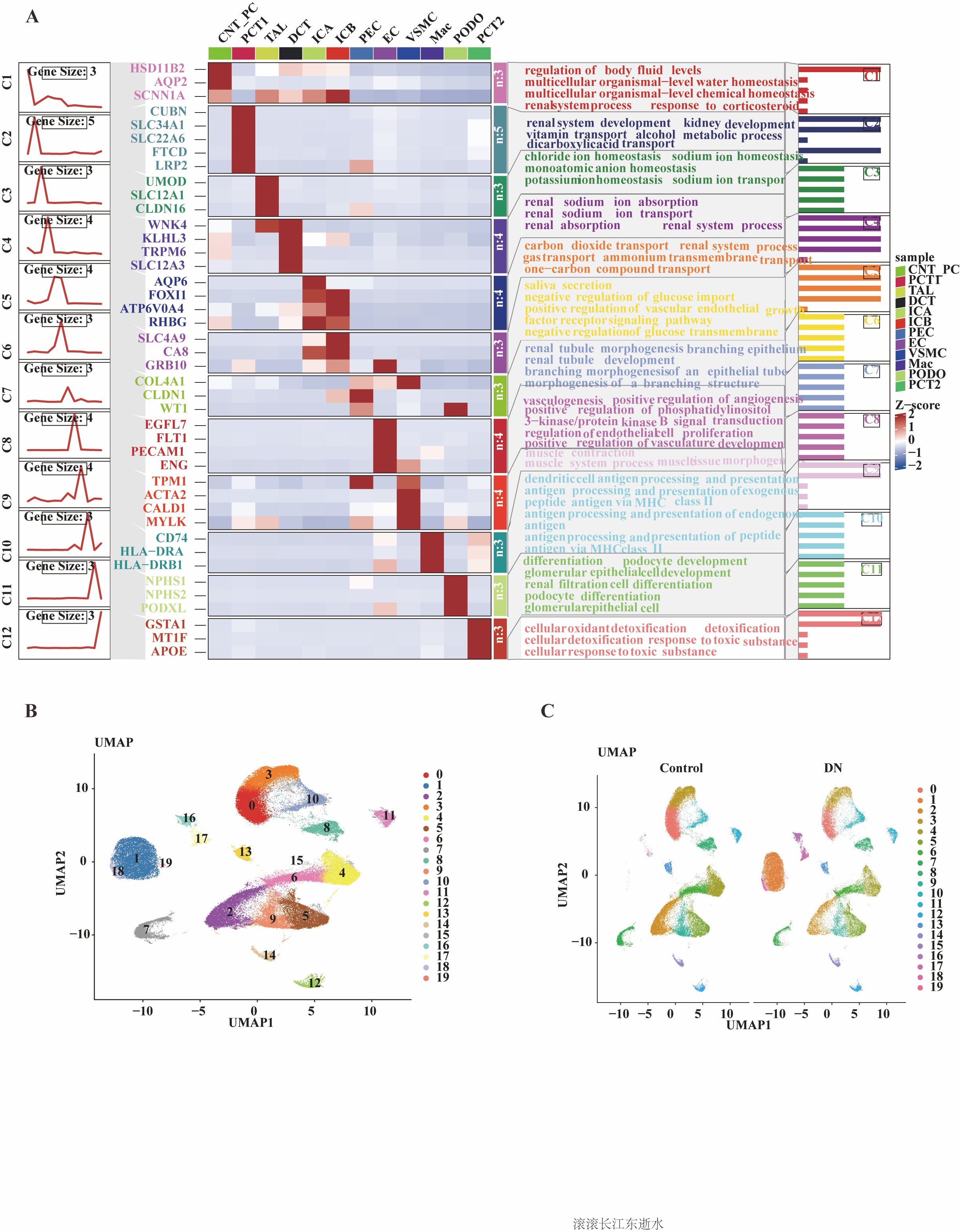

Supplement: Supplementary file 5 [file Image5.tif]

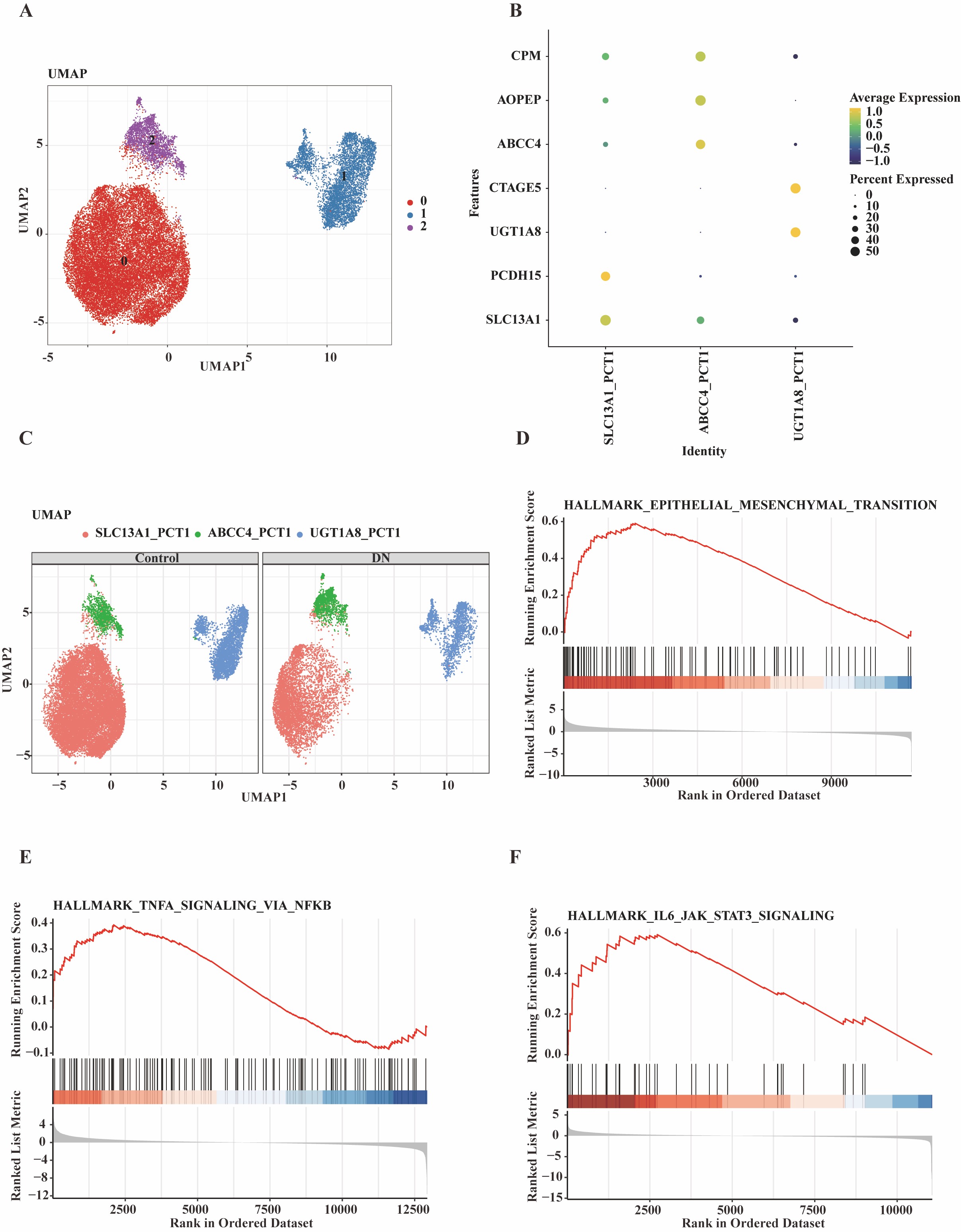

Supplement: Supplementary file 6 [file Image6.tif]
